# Computational mapping of productive POI–E3 ligase conformations to guide de novo degrader design: application to WEE1 and PKMYT1 PROTACs

**DOI:** 10.1186/s13321-026-01268-5

**Published:** 2026-07-24

**Authors:** Husam Nassar, Matthias Schmidt, Hany S. Ibrahim, Dina Robaa, Wolfgang Sippl

**Affiliations:** Department of Medicinal Chemistry, Institute of Pharmacy, Martin-Luther University Halle-Wittenberg, Halle (Saale), Germany

**Keywords:** PROTACs, Targeted protein degradation, Ubiquitination, WEE1, PKMYT1

## Abstract

Targeted protein degradation has emerged as a promising therapeutic strategy, yet rational degrader design remains challenged by the dynamic nature of protein of interest (POI)–E3 ligase interactions. While X-ray crystallography and cryo-EM provide valuable structural snapshots, they are insufficient for capturing the conformational heterogeneity underpinning efficient ubiquitination and degradation. Here, we present a unified computational workflow to systematically generate and evaluate POI–E3 ligase conformational states for CRBN- and VHL-mediated proteolysis-targeting chimeras (PROTACs). The workflow integrates warhead connectivity analysis, conformational clustering, ubiquitination accessibility assessment and molecular dynamics simulations to identify productive POI–E3 ligase geometries. Analysis of experimental structures revealed that PROTAC linkers do not exceed 15 Å, providing a practical attachment-atom distance based filter for docking-derived models. Furthermore, POI–E3 ligase conformations differing by more than 7.5 Å Cα RMSD exhibited distinct ubiquitination profiles, offering quantitative guidance for defining structurally and functionally divergent states. Experimental ternary complexes consistently positioned one or more solvent-exposed POI lysine residues within 50 Å of the E2 catalytic Cys111, establishing a mechanistically grounded criterion for ubiquitination competence. Validation against 34 experimentally determined PROTAC ternary complexes achieved a 97% recovery rate and identified multiple ubiquitination competent conformations beyond experimental snapshots. The workflow was subsequently applied to model productive WEE1-CRBN and PKMYT1-CRBN conformations, for which no experimental structures are available. PROTAC induced-fit docking demonstrated that active PROTACs selectively engage productive POI–E3 ligase geometries with linker-compatible attachment atom distances. Overall, this study provides a quantitative, structure-based framework for guiding the rational design of ubiquitination-based degraders. The code and example data supporting this workflow are openly available at https://github.com/Husam-PSE/PROTACMap.

**Scientific contribution**

This study offers a generalizable computational framework for identifying productive POI–E3 ligase conformations. It demonstrates that effective degradation depends on the interplay between conformational diversity, feasible warhead connectivity and preserved ubiquitination competence, rather than solely on ternary complex stability or binding affinity. Our computational approach was applied on WEE1 and PKMYT1 PROTACs for which no experimental ternary structure is available.

**Graphical Abstract:**

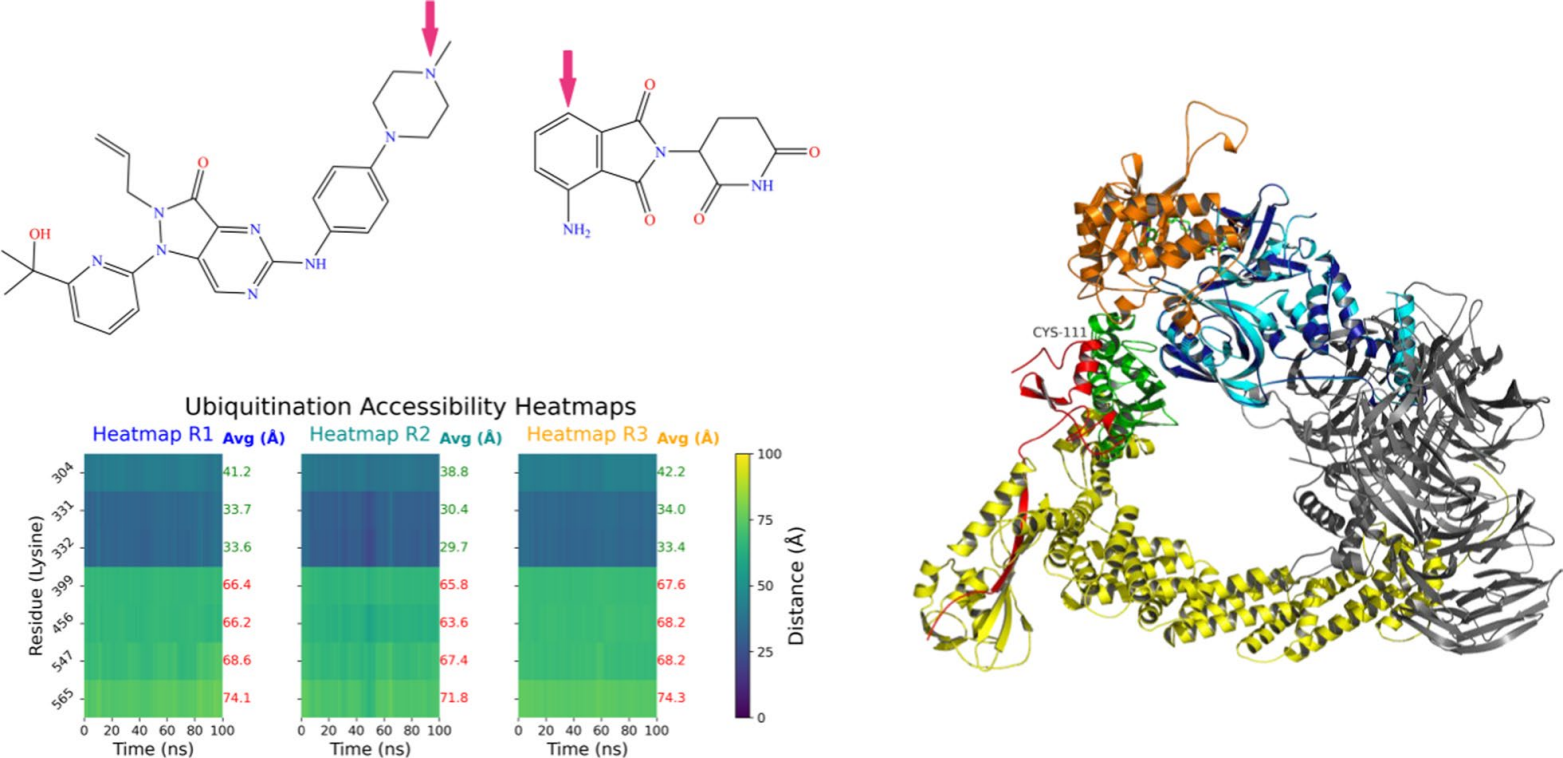

**Supplementary Information:**

The online version contains supplementary material available at 10.1186/s13321-026-01268-5.

## Introduction

Targeted protein degradation has emerged as an attractive strategy in the development of next-generation therapeutics [1]. Among these modalities, proteolysis-targeting chimeras (PROTACs) represent a novel therapeutic class that induces catalytic degradation of a target protein of interest (POI) through the ubiquitin–proteasome system. PROTACs are heterobifunctional molecules consisting of a POI-binding warhead and an E3 ligase-recruiting warhead connected by a linker [2–4]. The most commonly recruited E3 ligases in PROTAC design are cereblon (CRBN) and von Hippel-Lindau (VHL), both of which function as substrate-recognition subunits of Cullin-RING ligase (CRL) ubiquitination systems [5, 6]. Successful POI degradation depends critically on the structural organization of the POI–PROTAC–E3 ligase ternary assembly that enables efficient ubiquitin transfer [7]. Ubiquitin serves as a signal for proteasomal recognition and is transferred from the E2 ubiquitin-conjugating enzyme, recruited by the CRL complex, to solvent-exposed lysine residues on the POI [8–11]. Variations in the relative orientation of the POI and the E3 ligase influence lysine positioning and accessibility to ubiquitination, which consequently affects the efficiency of PROTAC-induced degradation. Structural insights from CRL ubiquitination assemblies indicate that solvent-exposed lysine residues on the POI are ubiquitination-competent when located within approximately 50 Å of the catalytic E2 cysteine (Cys111) [12–14].

Several studies have demonstrated that the cooperativity and stability of PROTAC ternary complexes correlate with degradation efficiency. Long-lived ternary complexes often enhance degradation by increasing the residence time of the POI near the E2 ubiquitin-conjugating enzyme, which provides more opportunities for productive ubiquitin transfer [15–17]. However, ternary complex stability alone is insufficient to guarantee POI degradation. POI–E3 ligase interactions can adopt rigid conformations in which solvent-exposed lysine residues are inaccessible for ubiquitination, resulting in failed POI degradation [18]. Therefore, the ability of a PROTAC to induce a productive POI–E3 ligase conformation that enables efficient ubiquitin transfer is a fundamental prerequisite for degradation and is more critical than the formation of a highly stable ternary complex or high binding affinity to either individual binding partner [19]. While X-ray and cryo-EM structures have provided informative snapshots of PROTAC ternary complexes, these static structures capture only a single conformation within a highly dynamic landscape. As a result, they are inadequate for fully describing the conformational heterogeneity and transient geometries that underpin ubiquitination competence [20–23]. Computational modeling offers a powerful framework for systematically exploring the conformational space of POI–E3 ligase complexes beyond experimentally resolved structures. Protein–protein and PROTAC docking, combined with conformational clustering and molecular dynamics (MD) simulations, enable the identification of structurally diverse and functionally relevant ternary geometries [24–28]. Leveraging experimentally determined ternary complexes as structural benchmarks provides critical guidance for validating and refining computationally generated models [29–31]. Such integration ensures that de novo predictions remain grounded in experimentally observed binding modes while allowing exploration of previously uncharacterized conformational states. Nevertheless, further computational investigation is required to: (1) define the permissible warhead attachment atom distances that ensure physically feasible connectivity; (2) establish the extent of Cα RMSD variation at which distinct POI–E3 ligase conformations can be considered structurally and functionally divergent; and (3) determine how many ubiquitination-competent POI–E3 ligase conformations should be considered during rational PROTAC design.

**Fig. 1:**
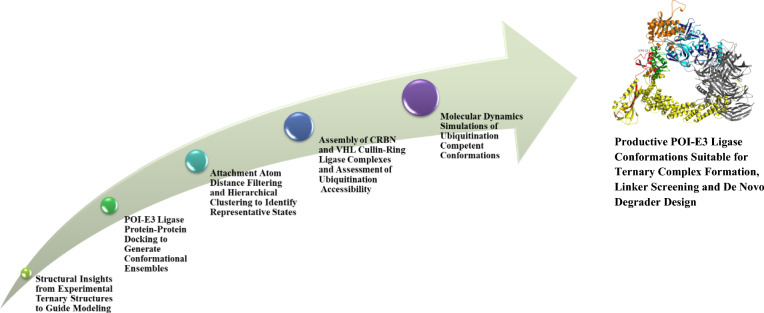
Overview of the integrative computational pipeline used to identify ubiquitination-competent POI–E3 ligase geometries

In this study, we present a unified computational workflow to generate productive POI–E3 ligase conformational ensembles across CRBN- and VHL-mediated PROTAC systems (Fig. 1). By integrating structural benchmarking, warhead connectivity and conformational clustering, ubiquitination accessibility assessment and MD simulations, we aim to identify productive ternary geometries and inform rational PROTAC design. Our modeling pipeline was validated using 34 experimentally determined ternary structures, achieving a 97% recovery rate, and was subsequently applied to map productive WEE1-CRBN and PKMYT1-CRBN conformations. WEE1 and PKMYT1 are protein kinases belonging to the WEE family and are considered compelling therapeutic targets in oncology due to their essential roles in cell cycle modulation and their deregulated activity in tumor cells [32, 33]. These two kinases were chosen as test cases to examine the robustness of the workflow in settings where experimentally determined ternary complex structures are not available. A previously reported set of active WEE1 and PKMYT1 PROTACs, together with an in-house collection of inactive compounds, was used to assess the ability of our modeling approach to rationalize PROTAC activity and guide future de novo degrader design.

## Methodology

### Benchmark dataset selection and input structure preparation

#### CRBN and VHL benchmark dataset and structural analysis

To validate our computational pipeline, a benchmark dataset of 34 experimentally determined ternary complex structures available in the Protein Data Bank (PDB) was assembled. These complexes encompass four POIs targeted by six CRBN-mediated PROTACs and 10 POIs targeted by 28 VHL-mediated PROTACs. The full list of benchmark ternary complexes, including PROTAC names, PDB identifiers, and relevant details is provided in Table 1. For each ternary complex, the linker length of the cognate PROTAC and the ubiquitination accessibility of solvent-exposed POI lysine residues were initially measured in the static structures.

**Table 1:** Summary of benchmark PROTAC-mediated ternary complexes and the corresponding POI and E3 ligase monomer structures used for modeling

E3 ligase	POI	PROTAC ID	Ternary complex PDB ID	Input monomers PDB ID	
				E3 ligase	POI
CRBN	BRD4BD1	dBET23	6BN7	8OIZ	3MXF
		dBET6	6BOY		
	BRD4BD2	CFT-1297	8RQ9		6DUV
	PTPN2	PROTAC1	8UH6		8U0H
	CDK2	Compound 4	9D0W		9D0U
		Compound 24	9NYR		
VHL	SMARCA2	PROTAC2	6HAX	5NVV	6HAZ
		PROTAC1	6HAY		
		ABCI1	7S4E		
		PROTAC5	7Z6L		7Z78
		PROTAC10	7Z76		
		PROTAC6	7Z77		
	SMARCA4	PROTAC1	8G1Q	5NVV	7TAB
	Bcl-xL	PROTAC6	6ZHC	5NVV	4QVX
		735b	8FY0		4QNQ
	BCL2	753b	8FY1	5NVV	4LVT
		WH244	8FY2		
	WDR5	PROTAC MS33	7JTO	5NVV	4QL1
		PROTAC MS67	7JTP		
		PROTAC PEG2	8BB2		
		PROTAC PEG1	8BB3		
		AD157	8BB4		
		AD122	8BB5		
	BRD4BD1	PROTAC9	7KHH	5NVV	3MXF
		PROTAC48	8BDS	8BDO	
		PROTAC49	8BEB	8BDO	
	BRD4BD2	MZ1	5T35	5NVV	6DUV
		AT7	7ZNT		
		PROTAC51	8BDT		
		PROTAC48	8BDX		
	KRas	PROTAC3	8QW6	5NVV	8QUG
		PROTAC4	8QW7		
	FAK	GSK215	7PI4	5NVV	6I8Z
	WEE1	AZD1775	8WDK	5NVV	5V5Y

To account for the dynamic behavior of ternary complexes, 1 μs MD simulations were performed on ternary complexes representing unique protein–protein conformations (MD settings are described in detail in section 5). For example, the SMARCA2-VHL conformation is experimentally resolved with six different PROTACs (PDB IDs: 6HAX, 6HAY, 7S4E, 7Z6L, 7Z76 and 7Z77). Structural comparison revealed that 6HAX, 6HAY, 7S4E and 7Z77 adopt the same SMARCA2-VHL conformation, so only 6HAX was simulated. In contrast, 7Z6L and 7Z76 exhibit distinct conformations and both were simulated to cover the structural diversity [34–36]. The structural insights derived from protein–protein conformational dynamics, attachment atom distances and long-term lysine ubiquitinability were then used to inform the development and parameterization of the modeling pipeline, such as attachment-atoms distance-based filtering and clustering thresholds, without biasing docking calculations.

#### Preparation of unbound POI and E3 ligase input structures

Protein three-dimensional structures were retrieved from the PDB and prepared using the Protein Preparation Workflow implemented in the Schrödinger Suite v2025. To generate input E3 ligase structures, two unbound CRBN and two unbound VHL monomers were selected. For CRBN, structures co-crystallized with pomalidomide (PDB ID: 8OIZ) and lenalidomide (PDB ID: 8RQA) were selected. For VHL, structures co-crystallized with Ligand 3 (PDB ID: 5NVV) and Compound 21 (PDB ID: 8BDO) were used. The following POIs were used as unbound monomeric input structures: BRD4BD1, BRD4BD2, PTPN2, CDK2, SMARCA2, SMARCA4, Bcl-xL, BCL2, WDR5, KRas, FAK and WEE1. The complete list of all POI and E3 ligase monomer structures, along with their corresponding PDB identifiers, is provided in Table 1. The selection of POI structure and either VHL or CRBN structures was dictated by the warheads present in the corresponding PROTAC, ensuring consistency between the warhead of the unbound monomers and the PROTAC-employed warhead. In case the warhead employed in the PROTAC is not crystallized with its corresponding monomer, it was docked using Glide module. For example, to model CDK2-CRBN conformation (PDB IDs: 9D0W and 9NYR), the CRBN-binding moiety used in the reported CDK2 PROTAC, ((*R*)-3-(4-fluoro-3,5-dimethyl-2-oxo-2,3-dihydro-1H-benzo[d]imidazol-1-yl)piperidine-2,6-dione), was docked into the CRBN structure (PDB ID: 8OIZ) to generate a compatible E3 ligase input structure. All protein structures were prepared by adding hydrogen atoms, rebuilding missing side chains and loops and removing crystallographic water molecules and ions [37]. Protonation states and tautomeric forms of ionizable residues were assigned using PROPKA at pH 7.0. Finally, restrained energy minimization was performed using the OPLS4 force field under default settings to relieve local strains [38, 39].

### Protein–protein docking to generate POI–E3 Ligase conformational ensembles

Protein–protein docking was performed to explore the conformational space of POI–E3 ligase interactions in the absence of a PROTAC molecule. Docking calculations were carried out using Method 4B implemented in the Molecular Operating Environment (MOE) software package (Chemical Computing Group, Montreal, Canada; v2019.01) [26]. All MOE protein–protein docking parameters were kept at their default values. In each system, the POI was defined as the ligand, while the E3 ligase (CRBN or VHL) was treated as the fixed receptor. The docking site was defined using residues located within 4.5 Å of the respective warhead in both proteins. Docking was performed using rigid-body refinement, allowing extensive rotational and translational sampling of the POI relative to the fixed E3 ligase. The protocol identifies hydrophobic surface patches on both proteins and performs multiple independent docking calculations for every pair of matching hydrophobic patches. The final docking ensemble is then constructed by combining the results of multiple independent searches, after removal of duplicate poses.

### Attachment-atoms distance filtering and conformational clustering

#### Filtering based on distance between warhead attachment atoms

All protein–protein docking solutions were exported as individual PDB files, with the fixed E3 ligase pose saved as a single reference structure and the POI structures saved as varying docking poses. For each docking solution, the distance between the predefined attachment atoms of the POI-binding warhead and the reference E3 ligase-binding warhead was measured using PyMOL through a custom Python script.

Based on structural analysis of experimentally resolved PROTAC ternary complexes, a distance threshold of 15 Å was selected to reflect physically feasible warhead connectivity. Docking solutions exhibiting attachment-atom distances greater than 15 Å were removed from the conformational ensemble, while poses satisfying this criterion were retained for further analysis. The filtered set of POI–E3 ligase conformations was subsequently subjected to hierarchical clustering to identify distinct representative geometries.

#### Hierarchical clustering of filtered conformational ensembles

The filtered set of POI–E3 ligase conformations was subjected to hierarchical clustering using the SciPy library in Python through a custom script. Each POI docking pose was combined with the fixed E3 ligase pose into a single complex and the Cα atoms coordinates of both proteins were used for alignment. Pairwise structural comparisons between complexes were performed using the Kabsch algorithm to calculate the RMSD of the aligned Cα atoms [40]. The resulting RMSD values were compiled into a symmetric distance matrix, which was used to construct a dendrogram representing the hierarchical relationships among conformations. At each step, the two most similar clusters are merged iteratively until all conformations are grouped, allowing identification of clusters of closely related protein–protein orientations.

A threshold of 7.5 Å was applied: conformations with RMSD ≤ 7.5 Å were considered structurally similar, whereas RMSD > 7.5 Å indicated distinct protein–protein geometries. This threshold was chosen based on observations during modeling, which demonstrated that conformations differing by more than 7.5 Å typically exhibited different ubiquitination competence profiles (Figures S1-S3). From each cluster, a single representative conformation was selected based on the MOE S docking score (more negative values indicate more favorable protein–protein binding).

### Modeling CRBN and VHL CRL assemblies and assessing ubiquitination accessibility

The ubiquitination systems of CRL4CRBN (CRBN-DDB1-Cullin4-Rbx1-UBE2D2) and CRL2VHL (VHL-Elongin B/C-Cullin2-Rbx1-UBE2D2) were modelled using PyMOL. The CRL4CRBN assembly was constructed by first superimposing different DDB1 protein conformations (PDB IDs: 3EI1, 3EI2, 3EI3 and 3EI4) onto the DDB1-Cullin4-Rbx1-UBE2D2 structure (PDB ID: 8B3I), followed by the integration of the CRBN subunit (PDB ID: 8OIZ) through alignment with the shared DDB1 subunit. The different conformations of DDB1 were evaluated to account for the flexibility of its β-propeller domains which influence the orientation of CRBN and, consequently, the positioning of the target protein within the ubiquitination assembly [41, 42]. While some conformations resulted in CRBN overlapping with the UBE2D2 and/or Cullin4 subunits, the conformation derived from PDB entry 3EI3 provided the most favourable CRBN positioning (Figure S4). The CRL2VHL assembly was generated by incorporating the Rbx1-UBE2D2 arm (PDB ID: 6TTU) into the VHL-Elongin B/C-Cullin2-Rbx1 complex (PDB ID: 8WDK) through structural alignment based on the Rbx1 subunit. Both assemblies were subsequently prepared and energy-minimized as described in Section 1.2. To evaluate the positional stability of CRBN, VHL and Rbx1-E2 arm, the modeled assemblies were simulated for 1µs (Figure S5). Both assemblies were then validated by assessing the static and dynamic ubiquitination accessibility of the experimental PROTAC ternary structures by monitoring the distance between the Cα atoms of POI lysine residues and UBE2D2 catalytic Cys111 residue. Lysine residues on the POI were identified using BIOVIA Discovery Studio Visualizer v2016 as those exhibiting solvent-exposed surface area greater than 25%. Each representative POI–E3 ligase complex obtained from clustering was then superimposed onto the corresponding E3 ligase (CRBN or VHL) within the ubiquitination assemblies. Complexes that showed POI overlapping with the ubiquitination machinery subunits were considered non-feasible and removed, while others were retained for subsequent MD simulations.

### Molecular dynamics simulations of ubiquitinable POI–E3 conformations

The dynamics of the retained POI–E3 ligase conformations was checked by performing three independent MD simulation runs of 100 ns. The Desmond simulation package was employed to set up the systems and run the MD simulations [43]. The systems were solvated using the TIP3P water model in a periodic orthorhombic box with a 15 Å buffer distance from the protein surface in all directions and neutralized with either Na⁺ or Cl⁻ ions [44]. To avoid the interference of ions in the dynamics of warhead’s solvent-exposed motifs, they were excluded from placement within 6 Å from warhead atoms. Prior to performing the production simulation, A force restraint of 0.5 kcal·mol⁻^1^·Å⁻^2^ was applied to the warhead attachment atoms to mimic the presence of a PROTAC linker. For all the simulation runs, the OPLS4 force field was utilized. Initially, solvent molecules and ions were energy-minimized while restraining the protein–ligand complexes, followed by minimization of the entire system. The system was then equilibrated in multiple stages for 12 ps per each stage: an initial NVT equilibration at a temperature of 10 K with small restraints, followed by NPT equilibration with gradual restraint removal. A final unrestrained NPT equilibration ensured system stability before initiating the simulation production run. A cutoff of 9 Å was used to smoothly truncate the Lennard-Jones interactions and short-range coulombic interactions. The Particle Mesh Ewald (PME) summation was used to calculate the long-range electrostatic interactions [45]. Finally, 100 ns simulation trajectories were carried out at a temperature of 300 K and a pressure of 1.01325 bar in the NPT ensemble using a Nose-Hoover chain thermostat and a Martyna-Tobias-Klein barostat [46, 47]. The simulation was performed using a 2 fs time step and trajectory frames were recorded every 50 ps for subsequent analysis. To ensure robustness and reproducibility, each simulation was performed in triplicate using a different random seed, confirming that the observed behaviors were not dependent on specific initial velocities or configurations. In the case of ternary complexes, simulations were extended to 1 μs, conducted in duplicate, with trajectory frames recorded every 250 ps and no restraints imposed on attachment atoms while keeping all other simulation parameters unchanged. For analysis, warhead conformations were extracted from trajectory frames and saved as SDF files to monitor the distance between attachment atoms throughout the simulation time. POI–E3 ligase conformations were extracted as PDB files and aligned onto the modeled ubiquitination assembly superposed by the E3 ligase using PyMOL. Binary complexes in which at least one solvent-exposed POI lysine residue consistently remained within 50 Å of the E2 catalytic Cys111 throughout the simulation were classified as productive conformations.

### Application to WEE1 and PKMYT1: CRBN PROTACs

The validated computational pipeline was subsequently applied to model productive protein–protein conformations for WEE1-CRBN and PKMYT1-CRBN systems. WEE1 in complex with AZD1775 (PDB ID: 5V5Y) was docked to CRBN prepared from PDB ID: 8OIZ, while PKMYT1 in complex with compound 41 (PDB ID: 8D6F) was docked to CRBN prepared from PDB ID: 8RQA. Protein–protein docking generated extensive conformational ensembles, which were filtered based on warhead attachment-atom distances, clustered by Cα RMSD and screened for ubiquitination accessibility within the CRL4CRBN assembly. Geometrically feasible conformations (non-clashing with ubiquitination machinery subunits) were subsequently subjected to triplicate 100 ns MD simulations. Conformations exhibiting sustained ubiquitination accessibility during these simulations were selected for PROTAC docking followed by 1 μs MD simulations of the resulting ternary complexes.

#### Induced fit docking and MD simulations of active PROTACs

Induced‐fit docking implemented in MOE v2019.01 was performed to assess the ability of the modeled WEE1-CRBN and PKMYT1-CRBN binary complexes to accommodate previously published active degraders. Six active WEE1 CRBN-mediated PROTACs namely, MA071, ZNL-02-012, ZNL-02-040, ZNL-02-047, ZNL-02-096 and TL12-186 in addition to the recently published PKMYT1 CRBN-mediated PROTAC (D16-M1P2) were docked into their respective POI–E3 ligase conformations after being prepared by the LigPrep tool [48–51]. Warhead atoms in each protein–protein complex were used to define the active site. Pharmacophore placement was used to position the docked poses by applying the generated pharmacophore as a filter on the final docked poses. Four pharmacophore features were assigned for the WEE1 or PKMYT1 warhead scaffolds and four for the CRBN ligands with a sphere of 2 Å radius in each case. The pharmacophore features assigned to the WEE1 warhead, AZD1775, included one hydrogen bond donor and one hydrogen bond acceptor corresponding to the amine and the pyrimidine nitrogen of the aminopyrimidine hinge-binding scaffold, respectively. An additional hydrogen bond acceptor was assigned to the pyrazolone carbonyl oxygen, and an aromatic ring feature was defined by the pyridine moiety. For the PKMYT1 warhead (Compound 41), the pharmacophore model comprised a hydrogen bond donor and a hydrogen bond acceptor represented by the amide NH and carbonyl oxygen of the hinge-binding motif, respectively. In addition, an aromatic ring feature was assigned to the phenyl group, and a combined hydrogen bond donor/acceptor feature was attributed to the attached phenolic hydroxyl group. In both systems, the pharmacophore properties of the CRBN-binding ligands were defined by two hydrogen bond acceptors and one hydrogen bond donor corresponding to the two carbonyl oxygens and the imide nitrogen of the glutarimide moiety, respectively, along with an aromatic ring feature represented by the phenyl ring. MOE induced fit docking generated 1000 initial placement poses from which the top 100 poses based on the London dG scoring function were passed on to a refinement step. The refinement used the Generalized‐Born Volume Integral/Weighted Surface Area (GBVI/WSA) scoring function to retain the final 50 poses. GBVI/WSA is a force field‐based scoring function that determines the binding free energy (kcal/mol) of the docked compound from a given pose [52, 53].

#### Linker conformational search and PROTAC design guidance

Linker conformational searches were performed for the linkers of experimentally active WEE1 and PKMYT1 PROTACs, as well as linkers of in-house inactive PKMYT1 PROTACs (HI100, HI101, HI102 and HI103), which were used as negative controls. Warhead attachment atoms were added to each linker to account for the bond length between the linker terminal atoms and the corresponding warhead attachment points. To ensure compatibility with the D16-M1P2 linker, the PKMYT1 warhead (compound 4) and the CRBN warhead (D16) were docked into the modeled PKMYT1-CRBN conformations using Glide. The new complexes were also subjected to 100 ns MD simulations, during which the distances between the warhead attachment atoms were monitored.

Linker conformational ensembles were generated using ConfGen tool implemented in the Schrödinger Suite v2025. For each linker, up to 999 conformers were generated, and no force-field minimization was applied to the output conformers in order to preserve the raw geometric diversity of sampled linker states [54]. For each linker conformational ensemble, linker lengths were measured as the distance between the predefined attachment atoms using PyMOL. The resulting linker length distributions were then compared against the attachment-atom distance windows obtained from the WEE1-CRBN and PKMYT1-CRBN models to assess linker compatibility and to inform future PROTAC synthesis.

### Synthesis of PROTACs HI100-103

Chemical synthesis and analytical data of PROTACs HI100-103 were reported in details in the supplementary materials.

## Results

### Structural insights from experimental ternary structures as reference for modeling

To establish a structural reference set for subsequent modeling studies, we started by examining structural features of 34 experimentally resolved CRBN- and VHL-mediated PROTAC ternary structures. Analysis of the static experimental structures revealed substantial variability in linker lengths across the PROTACs. The shortest linker was observed in the FAK-VHL ternary complex mediated by GSK215 (PDB ID: 7PI4), with a linker length of 2.49 Å, whereas the longest linker was found in the BRD4BD2-CRBN complex mediated by CFT-1297 (PDB ID: 8RQ9), measuring 13.49 Å. For systems in which multiple PROTACs with distinct linker chemistries but identical attachment points were crystallized in the same POI–E3 ligase conformation, linker lengths were generally convergent. A notable exception was observed for WDR5-VHL–mediated PROTACs, which exhibited substantial variation in linker length despite engaging the same attachment atoms within an identical WDR5-VHL conformation. For example, BRD4BD1 VHL-mediated PROTACs, PROTAC48 and PROTAC49 (PDB IDs 8BDS and 8BEB) displayed similar linker lengths of 8.94 Å and 8.49 Å, respectively. Likewise, BRD4BD2 VHL-mediated PROTACs PROTAC51 and PROTAC48 (PDB IDs 8BDT and 8BDX) showed comparable linker lengths of 7.61 Å and 7.88 Å, respectively. In contrast, WDR5-VHL PROTACs spanned a much broader range, with linker lengths varying from 6.87 Å for PROTAC AD157 (PDB ID 8BB4) to 13.76 Å for PROTAC PEG2 (PDB ID 8BB2), despite connecting the same attachment atoms in the same WDR5-VHL geometry (Table S1, Supplement). To assess whether the experimental conformations are compatible with ubiquitin transfer and to validate the modeled ubiquitination assemblies, we next tested ubiquitination accessibility for each static experimental structure. For all 34 ternary complexes, at least one solvent-exposed lysine residue on the POI was located within 50 Å of the catalytic E2 Cys111 of the corresponding ubiquitination machinery (Table S2, Supplement).

**Fig. 2:**
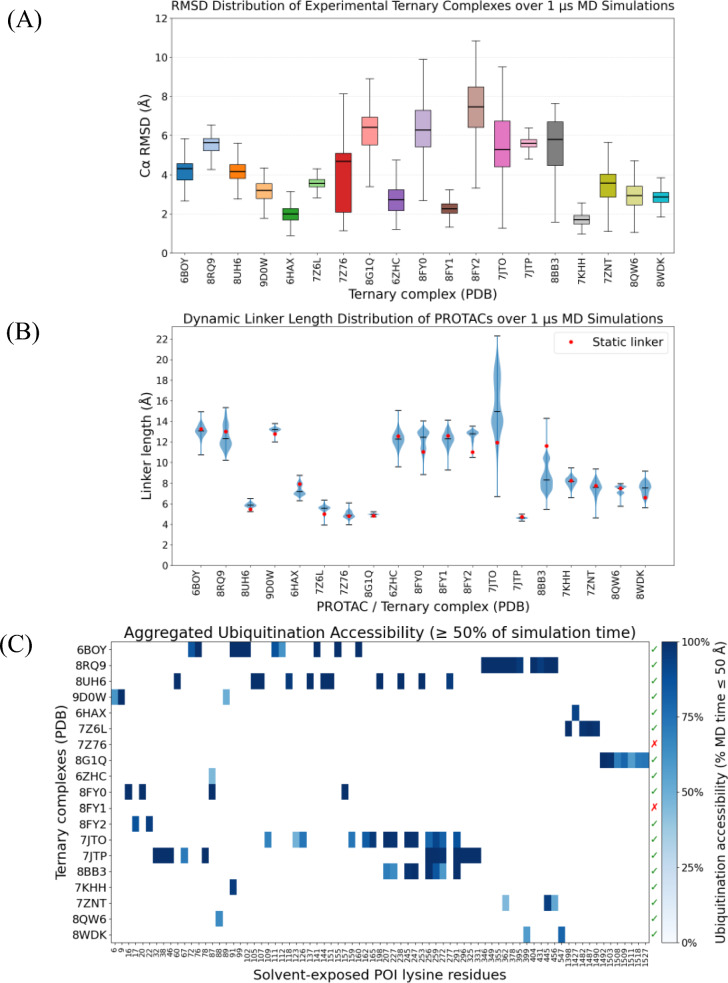
Dynamic and mechanistic insights derived from experimental ternary structures. **A** General dynamic behavior of the POI–E3 conformations, depicted as median Cα RMSD values along with the full RMSD range for each 1 µs MD trajectory. **B** Conformational behavior of PROTAC linkers over the 1 µs MD trajectories. **C** Aggregated ubiquitination accessibility heatmap, summarizing solvent-exposed POI lysine residues that remained within 50 Å of E2 Cys111 for more than 50% of simulation time

To evaluate the dynamic behavior of the experimentally resolved PROTAC ternary complexes, we performed 1 µs MD simulations for 19 representative CRBN- and VHL-mediated systems. Individual plots of Cα RMSD of the protein–protein complex, ubiquitination accessibility and linker length as a function of time for each ternary complex are provided in Supplementary material (Figures S6-S8, Supplement) for detailed inspection of system-specific and time-dependent dynamics. Figure 2A summarizes the general behavior of these complexes by depicting the median Cα RMSD of each trajectory, along with the RMSD range, providing a quantitative measure of the overall dynamics throughout the MD simulations. Across all experimental ternary complexes, median Cα RMSD values varied between 1.5 and 7.5 Å. CRBN-mediated ternary complexes exhibited a moderately stable behavior, with RMSD values maintained between approximately 3 Å and 6 Å throughout the simulation trajectories. However, VHL-mediated ternary complexes displayed greater heterogeneity in their dynamical profiles. Several systems corresponding to PDB IDs 6HAX and 7Z6L (SMARCA2), 6ZHC (Bcl-xL), 8FY1 (BCL2), 7KHH (BRD4BD1), 7ZNT (BRD4BD2), 8QW6 (KRas) and 8WDK (WEE1) maintained highly stable conformations, with RMSD values ranging between 1 and 4 Å. A second subset of VHL-mediated complexes, including PDB IDs 7Z76 (SMARCA2), 7JTO, 7JTP, and 8BB3 (WDR5), exhibited slightly higher RMSD values fluctuating between 4 and 6 Å. Notably, substantial conformational instability was observed for a small group of VHL-mediated ternary complexes, including PDB IDs 8FY0 (Bcl-xL), 8FY2 (BCL2), 7Z77 (SMARCA2) and 8G1Q (SMARCA4). These systems displayed RMSD values exceeding 7.5 Å while, in some cases, stabilizing at nearly 10 Å and significantly deviating from their experimentally resolved protein–protein conformations. Overall, fifteen of the nineteen simulated complexes (5 CRBN- and 10 VHL- mediated systems) exhibited well-maintained ternary architectures with no large-scale conformational rearrangements over the 1 µs timescale.

In addition to complex dynamics, we next examined the conformational behavior of the PROTAC linkers during the 1 µs MD simulations (Fig. 2B). For each system, the linker length was monitored over time and compared to the corresponding static value observed in the experimental structure. Across most systems, linker lengths sampled during the simulations fluctuated within a window of approximately 2 Å, and absolute linker lengths rarely exceeded 15 Å, even in systems exhibiting elevated Cα RMSD values. A notable exception to this general trend was observed for the VHL-mediated PROTAC MS33 targeting WDR5 (PDB ID: 7JTO). In this system, the linker explored a broader conformational space and transiently reached lengths exceeding 15 Å, longer than the static value. Nevertheless, even in this case, the majority of the sampled linker conformations remained below the same upper bound (15 Å) observed in the other systems. This consistent upper limit together with the observed length fluctuations provided practical physical criteria for defining acceptable attachment atom distances in the downstream POI–E3 ligase conformational modeling. In addition to linker dynamics, we assessed long-term POI ubiquitination accessibility throughout the simulation trajectories by monitoring the distances between the Cα atoms of solvent-exposed POI lysine residues and the Cα atom of the catalytic E2 residue Cys111. Figure 2C summarizes the aggregated ubiquitination accessibility, showing lysine residues that remained within 50 Å of E2 Cys111 for more than 50% of the simulation time. For nearly all simulated ternary systems, POI–E3 ligase conformations maintained consistent ubiquitinability, with at least one POI lysine residue residing within 50 Å of the catalytic E2 cysteine for the majority of the trajectory. For example, in the BRD4BD2-CRBN system, all solvent-exposed lysine residues were positioned within the ubiquitination-competent range, whereas in the BRD4BD2-VHL system, only Lys362, Lys445, and Lys456 fell within the ubiquitinable distance window. However, two notable exceptions were observed. In the SMARCA2-VHL conformation corresponding to PDB ID 7Z76, SMARCA2 lysine residues, particularly Lys1398, remained within 50 Å of E2 Cys111 for most of the first quartile of the simulation time; however, over the full trajectory, the distance slightly increased, yielding an overall average of 51.8 Å. Additionally, BCL2-VHL ubiquitinability was intermittent, with BCL2 Lys17 and Lys22 residing within 50 Å of the E2 Cys111 for only 7% and 16% of the simulation time, respectively.

### Generation and filtering of POI–E3 ligase conformational space

To explore the POI–E3 ligase conformational space of the 34 experimental PROTAC ternary complexes and to evaluate the ability of our modeling workflow to recover experimentally observed ternary arrangements, we generated large ensembles of POI–E3 complexes using protein–protein docking. The 34 ternary complexes comprise four POIs targeted by five CRBN-mediated PROTACs and 10 POIs targeted by 28 VHL-mediated PROTACs (Table1). For each system, POIs and E3 ligases were prepared to ensure the presence of the appropriate PROTAC warhead, either by selecting PDB structures already containing the warhead or by docking the warhead into the binding site using the Glide module. POI–E3 ligase docking was then performed using the protein docking tool implemented in MOE v2019, defining residues within 4.5 Å of each warhead as the interaction site. Importantly, docking was conducted in the absence of the PROTAC molecule and without imposing any linker-dependent constraints, allowing unbiased sampling of relative POI–E3 orientations. In addition, the input POI and E3 ligase structures used for docking were obtained from PDB entries in which each protein was solved independently in its unbound monomeric form, thereby avoiding bias from pre-existing POI–E3 interactions and more closely reflecting a scenario in which no PROTAC ternary complex structure is available.

**Fig. 3:**
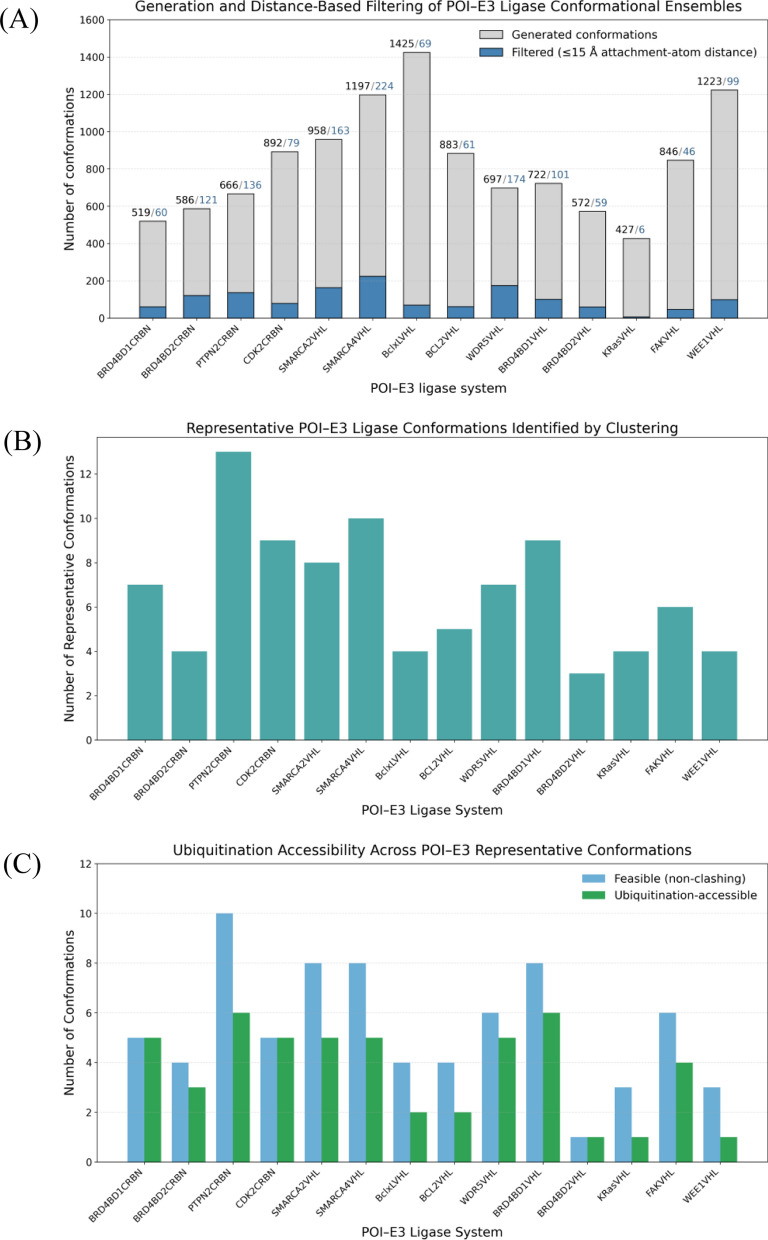
Conformational sampling of POI–E3 ligase complexes. **A** Docking of POI–E3 pairs generated hundreds to over a thousand conformations per system and attachment atom-based filtering identified conformations with feasible warhead connectivity. **B** Clustering of the docking ensembles dramatically reduced the conformational space, condensing it into a small number of representative conformations. **C** Ubiquitination accessibility analysis of the representative conformations categorized them into clashing conformations, which were excluded, but retained feasible geometries (blue bars) with conformations showing ubiquitinable lysines (green bars)

As shown in Fig. 3A, this approach generated hundreds to over one thousand POI–E3 conformations per system, reflecting extensive sampling of the conformational landscape. The total number of generated conformations ranged from ~400 to ~1400 poses, depending on the POI–E3 pair. VHL-mediated systems generally yielded larger conformational ensembles than CRBN-mediated systems. Notably, systems such as BCL-xL–VHL, SMARCA4-VHL and WEE1-VHL exhibited particularly large ensembles, exceeding 1000 generated conformations. To incorporate experimentally informed physical constraints derived from the static PROTAC ternary structures and their 1 µs MD simulations, the generated ensembles were subsequently filtered based on attachment atom distance. Specifically, POI–E3 ligase conformations exhibiting distances greater than 15 Å between the warhead attachment atoms were excluded, in accordance with the dynamic linker length upper bound observed across the experimental ternary complexes. This distance-based filtering resulted in a substantial reduction of the conformational space, retaining between ~1 and ~25% of the initially generated poses, depending on the system. Despite this reduction, all systems preserved a diverse set of POI–E3 conformations exhibiting geometrically feasible connectivity compatible with experimentally observed and dynamically sampled linker geometries.

The filtered POI–E3 ligase conformational ensembles provided initial docking samples with a wide range of physically realistic binding orientations ready for downstream analyses of POI–E3 conformational clustering.

### Clustering identifies distinct representative conformational states

Following attachment atom distance-based filtering, the remaining POI–E3 ligase conformational ensembles were clustered to identify distinct and representative protein–protein geometries. Clustering was performed using a Cα RMSD threshold of 7.5 Å, whereby conformations with RMSD ≤ 7.5 Å were considered structurally similar and grouped into the same cluster, while conformations differing by more than 7.5 Å were treated as distinct binding modes.

From each cluster, a single representative conformation was selected based on the MOE S docking score, with more negative values indicating more favorable protein–protein interactions. This procedure ensured that the retained representatives captured both geometric diversity and energetically favorable binding modes. As shown in Figure 3B, clustering resulted in a dramatic reduction of the conformational space for all modeled systems. Whereas the initial docking generated hundreds to thousands of POI–E3 ligase poses per system, clustering condensed these ensembles into a small number of representative conformations. Number of clusters ranged between 3 and 13 per system, with PTPN2-CRBN displaying the highest number, reflecting a comparatively diverse set of physically plausible POI–E3 orientations. These representative states captured the dominant binding geometries of each system and formed the basis for subsequent evaluation of ubiquitination competence.

### Ubiquitination accessibility assessment reveals multiple productive geometries

The representative POI–E3 ligase conformations identified through clustering were subsequently evaluated for ubiquitination accessibility to determine which geometries are compatible with productive ubiquitin transfer. For each system, the representative POI–E3 ligase conformations were aligned onto their corresponding CRL4CRBN or CRL2VHL ubiquitination assemblies by superposition of the shared E3 ligase subunits, thereby positioning the POI relative to the full ubiquitination machinery in a biologically relevant architecture.

As summarized in Fig. 3C, this analysis revealed ubiquitination competence across several representative conformations within the same POI–E3 ligase system. First, representative poses in which the POI sterically clashed with components of the ubiquitination machinery were excluded, retaining only geometrically feasible (non-clashing) POI–E3 ligase conformations. The number of feasible conformations varied across systems, ranging from as few as one to three (e.g., BRD4BD2-VHL, KRas-VHL and WEE1-VHL systems) to eight to ten in systems including PTPN2-CRBN, SMARCA2-VHL, SMARCA4-VHL and BRD4BD1-VHL. Among the retained feasible assemblies, multiple distinct conformations satisfied the productive ubiquitin transfer geometries by the presence of at least one solvent-exposed POI lysine residue within 50 Å of the catalytic E2 residue Cys111. Conversely, a small subset of non-clashing POI–E3 ligase conformations displayed POI lysine residues positioned beyond the 50 Å distance threshold from the E2 Cys111, rendering them non-ubiquitinable despite satisfying steric and linker-length criteria. Most systems retained multiple ubiquitinable conformations, with PTPN2-CRBN and BRD4BD1-VHL exhibiting the highest number (six conformations each), indicating that productive ubiquitination can arise from multiple distinct POI–E3 ligase geometries. In contrast, other systems exhibited a more limited number of ubiquitinable conformations or, in some cases, only a single productive geometry as in the cases of BRD4BD2-VHL, KRas-VHL and WEE1-VHL systems.

All geometrically feasible cluster representatives identified through static ubiquitination assessment, irrespective of the lysine positions, were subsequently advanced to 100 ns MD simulations. These simulations were used to validate the sustained attachment-atom connectivity and ubiquitination accessibility over time, ensuring that only models maintaining productive conformations were retained for PROTAC ternary complex design.

### MD simulations demonstrate the productivity of multiple conformations, including experimental geometries

**Fig. 4:**
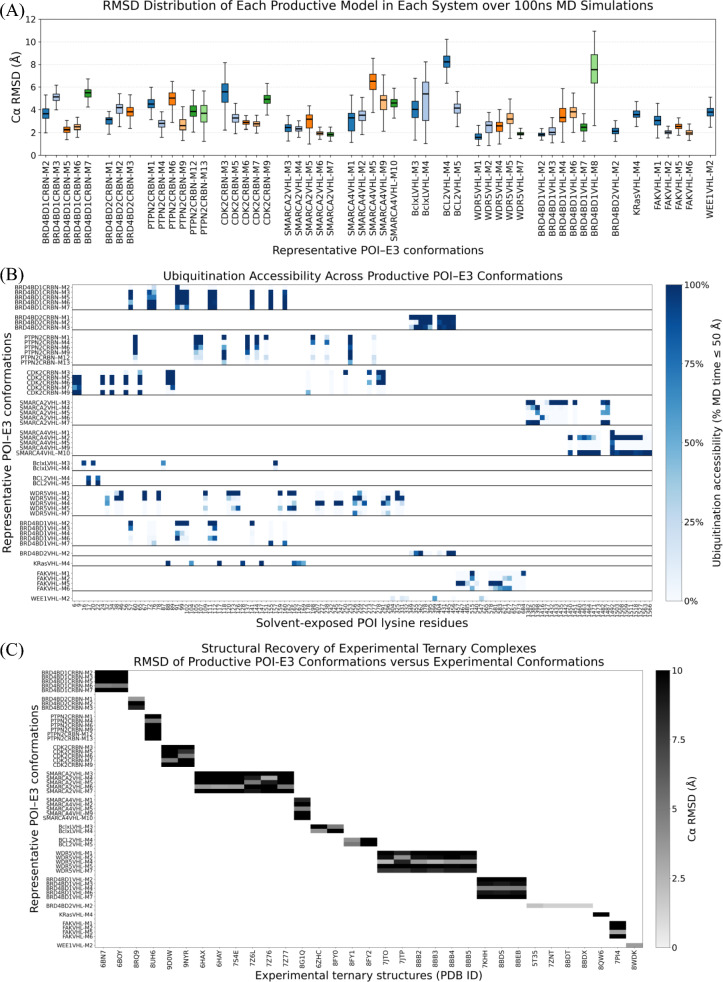
Dynamics, ubiquitination accessibility and structural validation of representative POI–E3 ligase models. **A** General dynamic behavior of the productive POI–E3 models, depicted as median Cα RMSD values along with the full RMSD range for each 100 ns MD trajectory. **B** Ubiquitination accessibility heatmap summarizing the percentage of simulation time during which solvent-exposed POI lysine residues remained within 50 Å of E2 Cys111. **C** Structural validation of productive modeled conformations by comparison with experimentally determined POI–E3 ligase ternary complexes, quantified as Cα RMSD relative to experimental structures

The representative POI–E3 ligase conformations identified through the conformational clustering and ubiquitinability assessment were subsequently advanced to MD simulations to evaluate their dynamics and sustained productivity. All feasible (non-clashing) conformations were simulated, regardless of the observed static ubiquitination accessibility, to capture potential conformational rearrangements that could affect lysine positions. For each conformation, 100 ns MD simulations were performed in triplicate using different random seeds to ensure robustness and reproducibility. Trajectory analyses primarily focused on Cα RMSD, warhead attachment atom distances and ubiquitination competence of solvent-exposed POI lysine residues. As summarized in Fig. 4A, the majority of POI–E3 ligase conformations exhibited RMSD distributions typically centered between 2 and 6 Å over the 100 ns simulations. These values reflect moderate flexibility while preserving the overall protein–protein geometries required for productive ternary complex formation. Two notable exceptions were observed. The BCL2-VHL–M4 model exhibited elevated RMSD values of approximately 8 Å and stabilized in a conformation different from the initial static structure. Similarly, the BRD4BD1-VHL–M8 complex showed pronounced RMSD fluctuations, reaching values above 10 Å, and underwent substantial conformational rearrangements.

Ubiquitination accessibility was then assessed by monitoring the distances between solvent-exposed POI lysine residues and the E2 catalytic Cys111. All non-clashing conformations in which POI lysine residues were outside the ubiquitinability range (>50 Å from E2 Cys111) remained highly stable; consequently, no conformational rearrangements were observed to reposition these lysine residues to be within the ubiquitination range. These models were considered non-productive and were excluded from further analysis. However, with the exception of BRD4BD1-VHL–M8, all other models retained the same solvent-exposed lysine residue observed in the static assessment within 50 Å of Cys111 for a substantial fraction of the simulation time and were therefore considered productive. Visual inspection of the BRD4BD1-VHL–M8 trajectories revealed significant conformational rearrangements that led to steric clashes between BRD4BD1 and components of the ubiquitination machinery leading to its classification as non-feasible. The sustained ubiquitinability of productive geometries is further illustrated in the ubiquitination accessibility heatmap (Fig. 4B), which summarizes the percentage of simulation time during which individual solvent-exposed POI lysine residues remained within 50 Å of E2 Cys111. Across systems, productive models typically maintained POI lysine residues within this distance threshold for a large fraction of the trajectory, often exceeding 50-75% of the simulation time.

Finally, the ability of the productive modeled conformations to recover experimentally observed POI–E3 ligase geometries of ternary complexes was evaluated by computing Cα RMSD values relative to the experimental structures (Fig. 4C). In all systems except KRas-VHL, at least one modeled productive conformation exhibited close structural similarity with the experimental reference, typically within 1-4 Å RMSD. In the case of KRas-VHL system, the only identified productive model (M4) showed Cα RMSD of 12.00 and 11.70 Å relative to the experimental conformations of PDB IDs 8QW6 and 8QW7, respectively. The recovery of 33 out of the 34 experimental structures confirms that the computational workflow not only captures physically feasible and ubiquitination-competent assemblies but also ensures sampling of experimentally identified POI–E3 ligase conformations. However, additional sampling may identify further ubiquitination-competent conformations and potentially improve structural recovery, particularly for the KRas-VHL system. Detailed modeling analyses of each individual system are provided in the Supplementary Information for in-depth inspection of Cα RMSD behavior, warhead attachment atom distances and ubiquitination accessibility trends across all productive models (Figures S9-S38 and Tables S3-S16, Supplement).

On the basis of the robustness and consistency of this workflow, it was subsequently applied to model WEE1-CRBN and PKMYT1-CRBN conformations. The aim of this step was to rationalize the activity of published and in-house WEE1- and PKMYT1-targeting CRBN-mediated PROTACs, to inform the future design of degraders targeting these kinases and to assess the generalizability of our computational workflow for modeling POI–E3 ligase conformations in the absence of experimental structural data.

### Modeling of productive WEE1-CRBN and PKMYT1-CRBN conformations

**Fig. 5:**
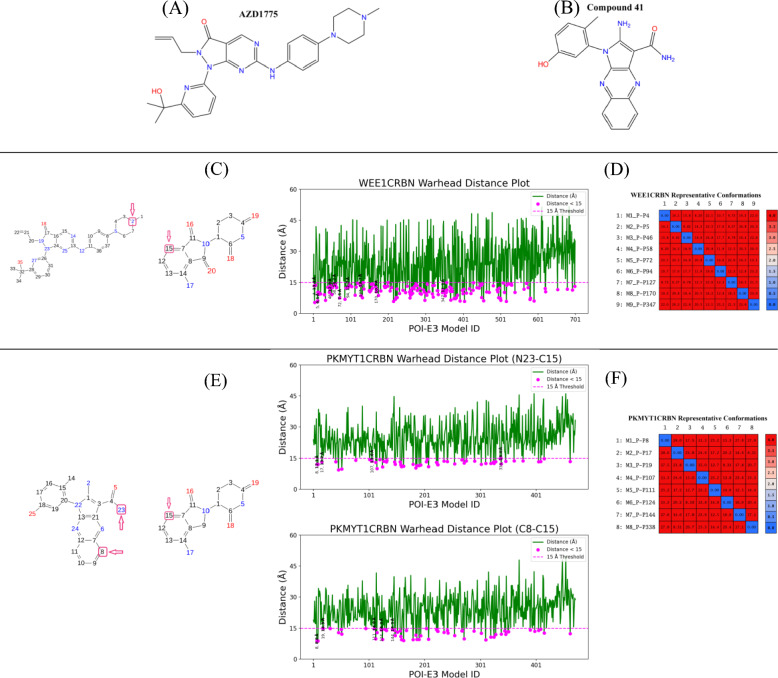
Docking, filtering, and clustering of WEE1-CRBN and PKMYT1-CRBN complexes. **A** 2D structure of WEE1 bound warhead, AZD1775. **B** 2D structure of PKMYT1 bound warhead, Compound41. **C** Protein–protein docking of WEE1 bound to AZD1775 (PDB ID: 5V5Y) against CRBN derived from PDB ID 8OIZ. **D** Pairwise Cα RMSD matrix of representative WEE1-CRBN models identified after clustering of the filtered ensembles. **E** Protein–protein docking of PKMYT1 bound to compound 41 (PDB ID: 8D6F) against CRBN derived from PDB ID 8RQA. **F** Pairwise Cα RMSD matrix of representative PKMYT1-CRBN models identified after clustering of the filtered ensembles. Attachment atoms are encircled pink in the warhead 2D structures while models with physically feasible warhead connectivity (attachment atom distance ≤ 15 Å) are indicated by pink points in the distance plots

Following validation of our computational workflow on experimentally characterized structures, the same pipeline was applied to model productive protein–protein conformations for the WEE1- and PKMYT1-CRBN systems. These two kinases were selected as case studies since corresponding experimental ternary complex structures are unavailable. For the WEE1 system, WEE1 bound to AZD1775 (PDB ID: 5V5Y) was docked to CRBN prepared from PDB ID 8OIZ while for the PKMYT1 system, PKMYT1 in complex with compound 41 (PDB ID: 8D6F) was docked to CRBN derived from PDB ID 8RQA. The 2D structures of AZD1775 and compound 41 are depicted in Fig. 5A and B. This docking step yielded 701 WEE1-CRBN and 472 PKMYT1-CRBN initial protein–protein conformations, respectively (Fig. 5C–F). The resulting conformational ensembles were filtered using the same criteria established earlier. Docking poses exceeding the 15 Å attachment atom distance limit were excluded, and the remaining conformations with feasible warhead connectivity were clustered using the 7.5 Å Cα RMSD threshold to identify structurally distinct protein–protein arrangements.

Attachment atoms were selected based on their solvent-exposed positions, allowing appropriate connectivity for PROTAC design. For CRBN, the selected attachment atom was the C15 atom of the phenyl ring of the CRBN ligand (pomalidomide or lenalidomide). The attachment atom for the WEE1 ligand AZD1775 was defined as the N2 atom of the piperazine ring, whereas two potential attachment atoms were considered for the PKMYT1 ligand (compound 41), namely the N23 atom of the amide group and the C8 atom of the phenyl ring. This filtering step yielded 156 WEE1-CRBN and 78 PKMYT1-CRBN geometrically feasible conformations suitable for linker design, corresponding to approximately 22% and 16.5% of the initially generated ensembles, respectively. The PKMYT1-CRBN count was combined for filtering based on both attachment atom pairs. Subsequent clustering of these filtered ensembles resulted in nine WEE1-CRBN and eight PKMYT1-CRBN distinct representative conformations. The clustering statistics and protein–protein docking energy scores of the representative WEE1-CRBN and PKMYT1-CRBN conformations are summarized in Tables 2 and 3, respectively. For the WEE1-CRBN system, cluster 9 was the most populated, comprising 71 members and exhibited average pairwise Cα RMSD value of 5.35 Å. For the PKMYT1-CRBN system, clustering was performed independently for the two selected attachment atom pairs (N23–C15 and C8–C15). Filtering based on the N23–C15 distance resulted in four clusters, whereas filtering based on the C8–C15 distance yielded six clusters. In both cases, the largest clusters contained 30 and 31 conformations, exhibiting average pairwise RMSD values of 4.61 Å and 4.81 Å, respectively. Across clusters, representative docking energies spanned a broad range (− 35.07 to − 64.19 kcal/mol for WEE1-CRBN and − 34.05 to − 69.59 kcal/mol for PKMYT1-CRBN), indicating no clear correlation between docking energy and conformational sampling population, as energetically favorable representatives were identified across clusters irrespective of cluster size.

**Table 2:** Clustering statistics and docking energy scores of representative WEE1-CRBN conformations

Cluster ID	Size	Average pairwise RMSD	Representative WEE1CRBN conformation	Representative S docking score (Kcal/mol)
9	71	5.35	M7_P-P127	− 45.12
7	39	4.72	M1_P-P4	− 64.19
8	15	4.57	M6_P-P94	− 47.11
2	12	4.28	M5_P-P72	− 48.52
5	9	5.16	M2_P-P5	− 63.76
1	4	4.63	M8_P-P170	− 42.90
4	3	1.74	M3_P-P46	− 51.90
6	2	1.87	M4_P-P58	− 50.02
3	1	0.00	M9_P-P347	− 35.07

**Table 3:** Clustering statistics and docking energy scores of representative PKMYT1-CRBN conformations

Attachment atom IDs	Cluster ID	Size	Average pairwise RMSD	Representative PKMYT1CRBN conformation	Representative S docking score (Kcal/mol)
N23–C15	4	30	4.64	M1_P-P8	− 69.59
	3	24	4.07	M4_P-P107	− 49.08
	2	9	3.40	M8_P-P338	− 34.05
	1	3	5.73	M2_P-P17	− 63.25
C8–C15	6	31	4.81	M1_P-P8	− 69.59
	5	23	3.85	M4_P-P107	− 49.08
	3	14	4.21	M3_P-P19	− 62.56
	4	8	4.15	M6_P-P124	− 46.69
	1	1	0.00	M5_P-P111	− 48.54
	2	1	0.00	M7_P-P144	− 45.45

**Fig. 6:**
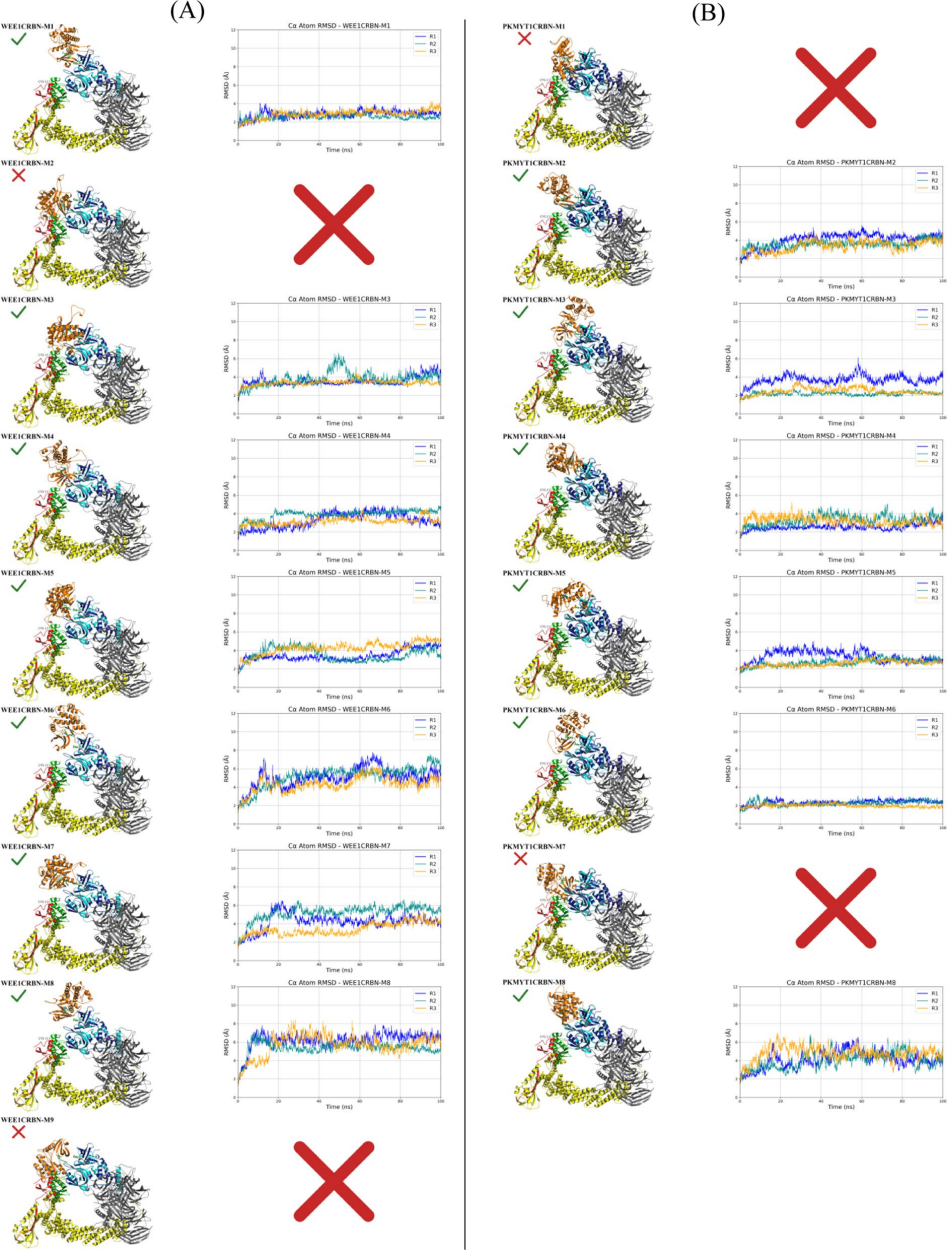
Dynamics of representative WEE1-CRBN and PKMYT1-CRBN complexes. **A** WEE1-CRBN and **B** PKMYT1-CRBN representative models incorporated into the modeled CRL4CRBN assembly (left) with corresponding Cα RMSD values from three independent 100 ns MD simulations (right)

**Fig. 7:**
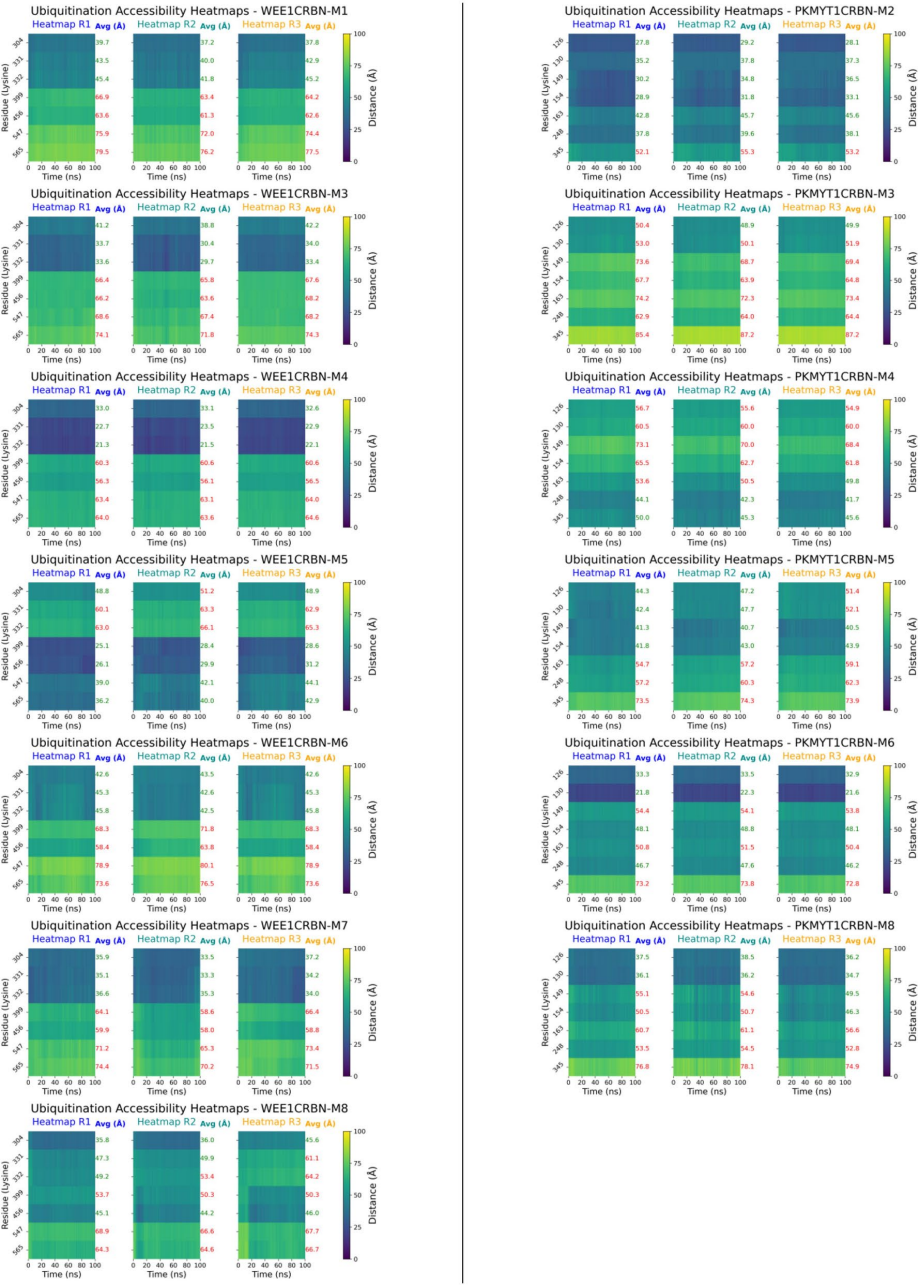
Ubiquitination accessibility of representative WEE1-CRBN and PKMYT1-CRBN complexes. **A** WEE1-CRBN and **B** PKMYT1-CRBN ubiquitination accessibility heatmaps summarizing the distances between Cα atoms of solvent-exposed lysine residues and E2 Cys111 throughout 100 ns MD simulations

The ubiquitination competence of the selected representative WEE1-CRBN and PKMYT1-CRBN models was next evaluated under static conditions by positioning each conformation within the CRL4CRBN ubiquitination assembly. Representative poses in which WEE1 or PKMYT1 exhibited steric clashes with components of the ubiquitination machinery were excluded, retaining only geometrically feasible, non-clashing conformations. This step resulted in the exclusion of two WEE1-CRBN models (WEE1-CRBN–M2 and WEE1-CRBN–M9) and two PKMYT1-CRBN models (PKMYT1-CRBN–M1 and PKMYT1-CRBN–M7). The remaining seven WEE1-CRBN and six PKMYT1-CRBN models were subsequently subjected to three independent 100 ns MD simulation runs to assess their dynamics and time-dependent ubiquitinability. As shown in Fig. 6, the retained representative models for both systems exhibited consistent and reproducible behavior across all three simulations. For the majority of models, the POI–E3 ligase conformations were well-maintained over the simulation timescale with Cα RMSD values stabilizing around 2-4 Å. Only WEE1-CRBN–M6 and M7, as well as PKMYT1-CRBN–M8 displayed moderate flexibility, with RMSD values fluctuating between 4 and 6 Å, yet without any large-scale conformational destabilization. In parallel, ubiquitination accessibility was evaluated dynamically by monitoring the distances between solvent-exposed lysine residues and the E2 Cys111 throughout the 100 ns simulations. As summarized in Fig. 7, all simulated models exhibited consistent and reproducible ubiquitination accessibility profiles across the three simulation runs, with at least one lysine residue remaining within 50 Å of E2 Cys111 for the whole duration of the simulations. In the WEE1-CRBN system, Lys304, Lys331 and Lys332 were ubiquitinable in five of the seven models, defining dominant and recurrent ubiquitination hotspots. WEE1-CRBN–M5 and WEE1-CRBN–M8 exhibited distinct accessibility patterns, demonstrated by the ubiquitinability of alternative lysine sets. In contrast, PKMYT1-CRBN models displayed greater variability and redundancy in accessible lysines, with most conformations exhibiting ubiquitinability through multiple distinct residues. Only PKMYT1-CRBN–M2 exhibited a single ubiquitination accessible lysine residue, Lys126. These ubiquitinable conformations were subsequently advanced to induced-fit docking of active PROTACs to assess their capacity to accommodate active PROTACs and form sustained, productive ternary complexes.

### Induced fit docking and stability of potent WEE1 and PKMYT1 PROTACs

To evaluate whether the productive conformations can accommodate active degraders, induced-fit docking was performed on the identified WEE1-CRBN and PKMYT1-CRBN models. Six reported CRBN-mediated WEE1 PROTACs (MA071, ZNL-02-012, ZNL-02-040, ZNL-02-047, ZNL-02-096, and TL12-186) and one CRBN-mediated PKMYT1 PROTAC (D16-M1P2, evaluated in both *R*- and *S*-oxa-azaspirodecane linker configurations) were included in this analysis (Table 4). Docked poses were required to satisfy the pharmacophoric features defined for the corresponding warheads in the modeled POI–CRBN conformations (Section 6.1; Figure S39, Supplement). Despite conformational diversity among the WEE1-CRBN models, all were capable of accommodating MA071, ZNL-02-012, ZNL-02-040 and ZNL-02-047, with the most favorable docking scores consistently observed in the WEE1-CRBN–M3 conformation (− 16.27, − 16.40, − 15.29 and − 15.63 kcal/mol, respectively). In contrast, ZNL-02-096 was exclusively accommodated in the WEE1-CRBN–M5 model with a docking score of -14.01 kcal/mol while docking of TL12-186 was only successful in WEE1-CRBN–M5 and WEE1-CRBN–M8 models with the superior docking score being in M5 (− 14.29 kcal/mol). WEE1-CRBN models accessibility to PROTACs and energy scores of successfully docked poses are summarized in Table 5. For the PKMYT1 system, ternary complex formation was more restrictive as docking of D16-M1P2 was only achieved in PKMYT1-CRBN–M4 conformation, irrespective of linker stereochemistry. The top-ranked pose of D16-M1P2, in both stereochemical forms of the oxa-azaspirodecane linker motif, showed docking scores of − 10.85 kcal/mol for the *R* configuration and − 10.70 kcal/mol for the *S* configuration.

**Table 4:** Reported CRBN-mediated WEE1 and PKMYT1 active PROTACs included in this study

PROTAC ID	2D Structure	Activity (nM)	References
MA071		Cell death (BT549) IC_50_ = 7.90	[48]
ZNL-02-012		Cell death (MOLT-4) IC_50_ = 7.28	[49]
ZNL-02-040		Cell death (MOLT-4) IC_50_ = 4.46	[49]
ZNL-02-047		Cell death (MOLT-4) IC_50_ = 3.18	[49]
ZNL-02-096		WEE1 degradation DC_50_ = 10.00	[49]
TL12-186		Cell death (MOLT-4) IC_50_ = 10.30	[50]
D16-M1P2		PKMYT1 degradation DC_50_ = 0.70	[51]

**Table 5:** Induced-fit docking energy scores of reported CRBN-mediated WEE1 PROTACs

WEE1-CRBN model	Docking Scores of Top-ranked Poses (Kcal/mol)					
	MA071	ZNL-02-012	ZNL-02-040	ZNL-02-047	ZNL-02-096	TL12-186
WEE1-CRBN-M1	− 15.97	− 14.98	− 13.20	− 14.46	NA	NA
WEE1-CRBN-M3	− 16.27	− 16.41	− 15.29	− 15.63	NA	NA
WEE1-CRBN-M4	− 15.61	− 14.88	NA	− 13.81	NA	NA
WEE1-CRBN-M5	− 15.40	− 15.73	− 15.19	− 14.80	− 14.01	− 14.29
WEE1-CRBN-M6	− 15.72	− 14.51	− 13.50	NA	NA	NA
WEE1-CRBN-M7	− 16.18	− 15.31	− 12.94	− 14.68	NA	NA
WEE1-CRBN-M8	− 15.79	− 15.04	− 13.91	− 13.82	NA	− 13.56

**Fig. 8:**
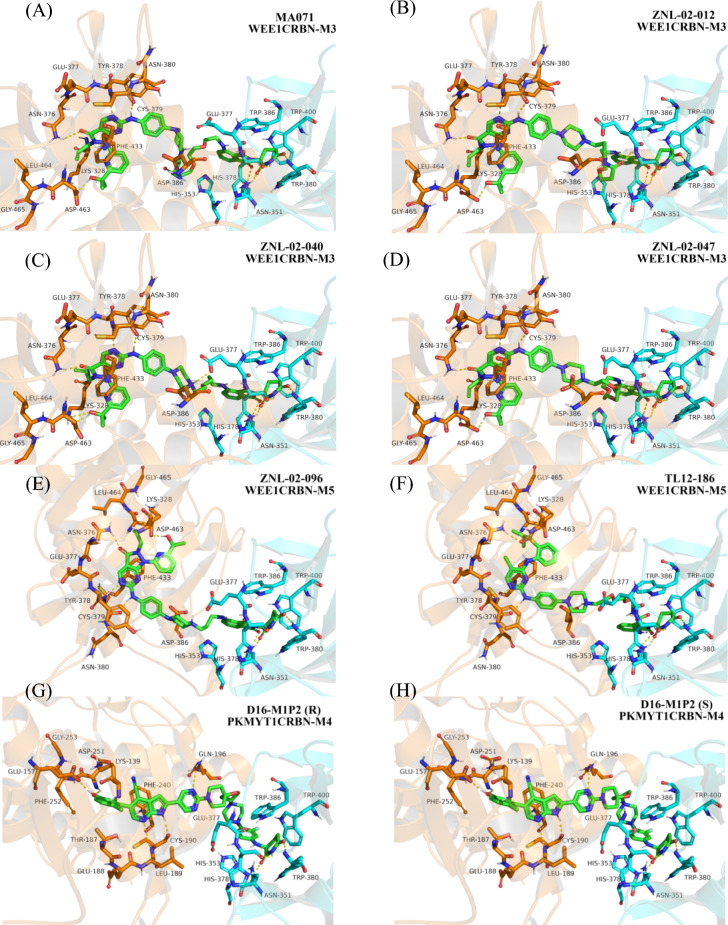
Binding modes of active PROTACs in representative WEE1-CRBN and PKMYT1-CRBN conformations. **A**–**D** MA071, ZNL-02-012, ZNL-02-040, and ZNL-02-047 in WEE1-CRBN–M3. **D**, **E** ZNL-02-096 and TL12-186 in WEE1-CRBN–M5. **F**, **G** Both stereoisomers of D16-M1P2 in PKMYT1-CRBN–M4. PROTACs are shown as green sticks, POI residues (WEE1/PKMYT1) as orange sticks, CRBN residues as cyan sticks and hydrogen bonds as yellow dashed lines

**Fig. 9:**
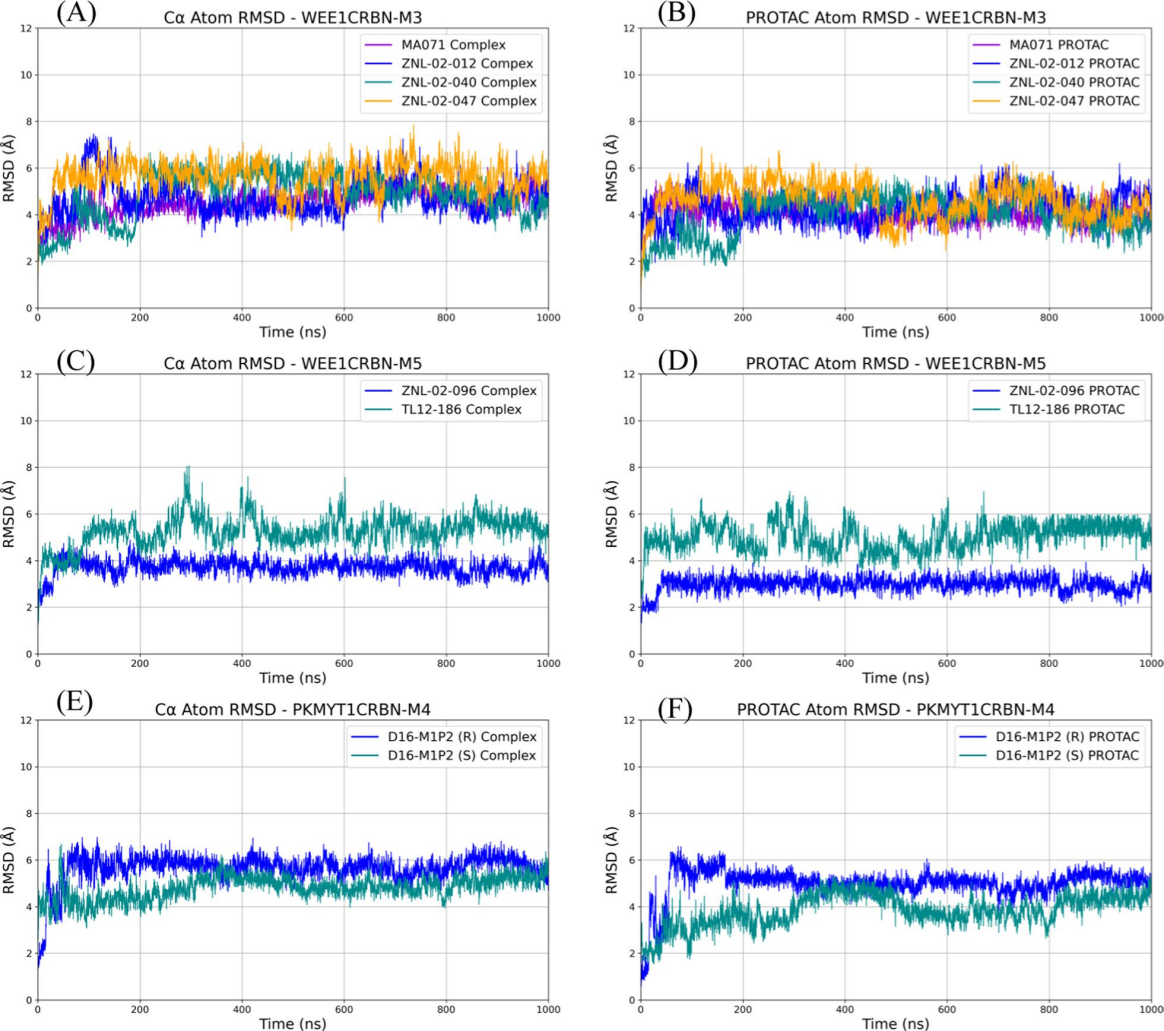
RMSD values of active PROTAC ternary complexes over 1 µs MD simulations. **A**, **B** MA071, ZNL-02-012, ZNL-02-040 and ZNL-02-047 in WEE1-CRBN–M3. (C,D) ZNL-02-096 and TL12-186 in WEE1-CRBN–M5. (E,F) Both stereoisomers of D16-M1P2 in PKMYT1-CRBN–M4. Left panels show Cα RMSD of proteins; right panels show PROTAC RMSD fitted on protein Cα

The top-ranked poses—MA071, ZNL-02-012, ZNL-02-040 and ZNL-02-047 in WEE1-CRBN–M3; ZNL-02-096 and TL12-186 in WEE1-CRBN–M5; and both stereochemical forms of D16-M1P2 in PKMYT1-CRBN–M4—showed successful alignment with the defined pharmacophores. In all cases, the PROTAC linkers adopted conformations embedded at the POI–CRBN interface, while preserving the key interactions of each warhead within its respective binding pocket (Figure 8). Subsequently, these successfully generated ternary complexes were advanced to extended 1µs MD simulations to assess their long-timescale dynamic behavior. Across all systems, the ternary complexes remained moderately stable throughout the simulations, with Cα RMSD values generally ranging between 4 and 6 Å and PROTAC RMSD values between 3 and 5 Å (Fig. 9). Notably, the ZNL-02-096–mediated WEE1-CRBN–M5 ternary complex exhibited the lowest overall structural deviations, stabilizing at 4 Å for Cα atoms and 3 Å for PROTAC atoms. In the TL12-186–mediated WEE1-CRBN–M5 ternary complex, three transient RMSD excursions reaching ~7-8 Å were observed; however, each persisted for less than 10 ns. Throughout all simulations, PROTAC-POI and PROTAC-CRBN interactions were well maintained, displaying moderate to high occupancy rates over time (Figures S40-S42, Supplement). Moreover, all ternary complexes exhibited sustained ubiquitination accessibility throughout the 1 µs simulations, fully consistent with the trends observed previously in the 100 ns MD runs, thereby confirming the long-timescale productivity of their geometries (Figures S43-S45, Supplement).

### Linker length distributions rationalize PROTAC activity and guide de novo PROTAC design

To assess whether the attachment-atom distance windows derived from productive POI–E3 ligase conformations could rationalize PROTAC activity and inform linker design, we analyzed the conformational sampling of linkers from the studied active WEE1- and PKMYT1-targeting PROTACs, together with four in-house PKMYT1 PROTACs that were found to be inactive degraders. 2D structures of PKMYT1 PROTACs (H100-103) are shown in Table 6 (Synthesis and analytical characterization of the four compounds are given in the Supplement, Schemes 1 and 2). For each compound, linker conformers were generated using the ConfGen tool implemented in the Schrödinger Suite (v2025), and their end-to-end distances were measured and directly compared with the attachment-atom distances observed during 100 ns MD simulations of the corresponding productive POI–E3 ligase models. Prior to this analysis, the warheads employed in the PKMYT1 PROTACs (Compound 4 and D6) were docked into the PKMYT1-CRBN models and subjected to triplicate 100 ns MD simulations to confirm their compatibility with the D16-M1P2 employed warheads. This step ensured that the attachment-atom distance windows extracted from POI–E3 simulations could be meaningfully applied to linker screening and rational PROTAC design based on Compound 4 and D6 structures.

**Table 6:** In-house CRBN-mediated PKMYT1 PROTACs

PROTAC ID	2D structure
HI100	
HI101	
HI102	
HI103	

**Fig. 10:**
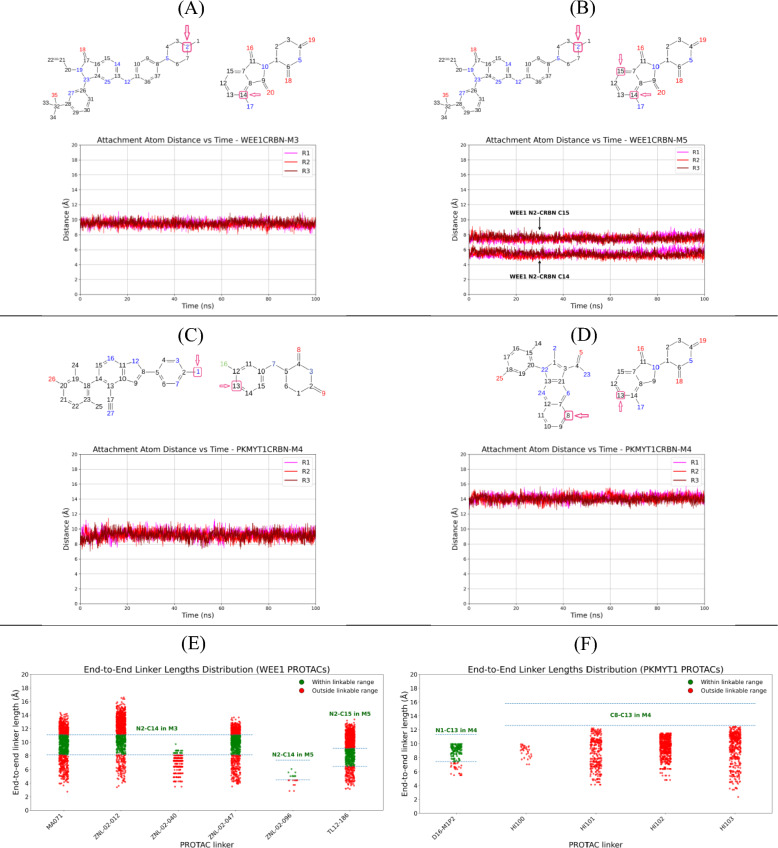
Attachment-atom distance compatibility between productive POI–E3 models and PROTAC linkers. **A** WEE1-CRBN–M3 attachment-atom distances for MA071, ZNL-02-012, ZNL-02-040 and ZNL-02-047 compounds. **B** WEE1-CRBN–M5 attachment-atom distances for ZNL-02-096 and TL12-186 compounds. **C** PKMYT1-CRBN–M4 attachment-atom distances for the D16-M1P2 compound. **D** PKMYT1-CRBN–M4 attachment-atom distances for in-house inactive compounds. **E** and **F** Linker length distributions of WEE1 and PKMYT1 PROTACs compared with the attachment-atom distance windows monitored in their corresponding models

For the WEE1-CRBN–M3 model, the attachment-atom distance defined by the AZD1775 N2- Pomalidomide C14 pair, corresponding to the link points in ZNL-02-012, ZNL-02-040, and ZNL-02-047, remained between approximately 8.5 and 10.5 Å throughout the simulations (Fig. 10A). In the WEE1-CRBN–M5 model, two distinct attachment-atom pairs were considered, reflecting the link points of ZNL-02-096 (N2-C14) and TL12-186 (N2-C15). In this case, the N2-C14 distance fluctuated between 5 and 7 Å, whereas the N2-C15 distance exhibited a range of 7 to 9 Å (Fig. 10B). For the PKMYT1-CRBN–M4 model, simulations performed with Compound 4 and D6 revealed attachment-atom distances (Compound 4 N1- D6 C13) ranging from approximately 8 to 11 Å (Fig. 10C). Simulations of the same model with Compound 41 and lenalidomide exhibited a longer attachment-atom distance (Compound 41 C8- Lenalidomid C13), fluctuating between 13 and 15 Å (Fig. 10D).

Analysis of the linker conformer length distributions demonstrated a strong correspondence between PROTAC activity and compatibility with the attachment-atom distance windows derived from productive POI–E3 models. All active WEE1 PROTACs displayed linker conformations whose lengths aligned with the monitored attachment-atom distances of their respective productive models (Figure 10E). Specifically, linker conformers of ZNL-02-012, ZNL-02-040, and ZNL-02-047 were compatible with the 8.5–10.5 Å distance window of WEE1-CRBN–M3. Notably, ZNL-02-096 linker conformers exhibited lengths that matched exclusively the N2–C14 distance window (5–7 Å) of WEE1-CRBN–M5, consistent with the observation that this was the only model capable of accommodating ZNL-02-096. Similarly, TL12-186 linker conformers aligned uniquely with the N2–C15 distance window (7–9 Å) of WEE1-CRBN–M5, and were also compatible with the WEE1-CRBN–M8 distance range (see Supplementary Material for a comprehensive comparison across models, Figures S46-S48). In the PKMYT1 system, D16-M1P2 linkers, in both stereochemical configurations, exhibited highly similar length distributions, each aligning well with the attachment-atom distance window observed for PKMYT1-CRBN–M4 (Fig. 10F). In contrast, the linkers of the inactive in-house PKMYT1 PROTACs (H100-H103), which connect C8 of Compound 41 to C13 of lenalidomide or pomalidomide, did not sample linker lengths compatible with any productive PKMYT1-CRBN C8–C13 attachment-distance window. These inactive linkers adopted lengths between 2 and 12 Å, whereas the shortest C8–C13 distance observed across all PKMYT1-CRBN models was approximately 13 Å, observed by the PKMYT1-CRBN–M4 model. Induced-fit docking of these inactive PROTACs was consistent with the observed linker lengths, as none of the models successfully accommodated them.

## Discussion

Recent advances in computational modeling have substantially improved our ability to characterize PROTAC-mediated ternary complexes, enabling structure-based exploration of degrader-induced protein–protein interactions [55–57]. Ensemble-based docking and MD simulations allow sampling of diverse POI–E3 conformational states beyond static experimental structures [58–60]. However, successful modeling of productive ternary complexes requires deeper incorporation of mechanistic constraints. In particular, physically feasible warhead connectivity, functional discrimination between conformational states, and ubiquitination accessibility are fundamental determinants of productive degradation that remain underrepresented in many current modeling workflows. Integrating these mechanistic insights is therefore essential for improving the predictive power of PROTAC ternary complex modeling and for guiding rational degrader design. In this study, we performed an integrated static and dynamic structural analysis of experimentally resolved ternary complexes to elucidate key geometric determinants underlying productive PROTAC-mediated ubiquitination and degradation. By monitoring linker connectivity, POI–E3 ligase conformational variation, ternary complex dynamics and ubiquitination accessibility, we derive quantitative structural thresholds that inform de novo degrader design.

Analysis of experimental ternary structures revealed that physically feasible warhead connectivity is tightly constrained, with lengths not exceeding 15 Å. This observation supports the use of a conservative 15 Å distance threshold to filter docking-derived POI–E3 conformations and exclude geometries unlikely to be realizable by a PROTAC molecule. MD simulations further showed that overall linker lengths fluctuate within a window of approximately 2 Å, indicating balanced conformational plasticity. Consistent with these observations, Yang et al. reported that degradation efficiency depends on the formation and maintenance of specific hydrophobic patches at the POI–E3 interface, which are sensitive to linker length and flexibility. Both insufficient and excessive linker flexibility were shown to reduce degradation efficiency by disrupting key interfacial hydrophobic interactions [61]. Our assessment of linker lengths further supports and extends the conclusion that physically feasible warhead connectivity and constrained linker flexibility dictate whether functionally relevant interfacial contacts can form, thereby controlling degradation outcomes [62, 63]. One notable exception was observed for WDR5-VHL–mediated PROTACs, which exhibited substantial linker conformational sampling. This pronounced linker flexibility observed in MD simulations is consistent with experimental structures, in which WDR5-VHL PROTACs display static linker lengths ranging from 6.9 to 13.8 Å, despite connecting the same attachment atoms within identical WDR5-VHL conformations [64].

Ubiquitination accessibility assessment further showed that experimental ternary geometries are productive, consistently position one or more solvent-exposed POI lysine residues within 50 Å of the E2 catalytic Cys111, and providing a mechanistically meaningful criterion for ubiquitination competence of modeled structures. Consistent with recent cryo-EM and ubiquitinomics analyses of the MZ1-induced BRD4BD2-VHL complex, our modeled CRL2VHL ubiquitination machinery correctly identified experimentally observed ubiquitination-competent lysines including Lys362, Lys368, Lys445 and Lys456 [10]. Although Lys368 was positioned within the ubiquitinable range, it was excluded in our analysis due to limited solvent accessibility. Notably, experimental ternary complexes displayed substantially different dynamics, with subsets showing high stability, moderate flexibility or pronounced instability. However, all complexes maintained the architecture required for sustained ubiquitination accessibility, indicating that high stability alone is insufficient to assess degradation outcomes. In light of these structural insights, we mapped productive POI–E3 ligase conformations of 34 experimental PROTAC ternary structures. The multiple productive POI–E3 ligase conformations we identified per system corroborate Ma et al.’s observation that the SMARCA2 domain occupies a broader range of productive positions relative to VHL than crystal structures alone suggest [65]. Furthermore, modeling of the CDK2-CRBN system yielded model M7_P-P197, which reproduces the conformation observed in the cryo-EM structure PDB 9D0W, and model M6_P-P103, which matches the recently resolved cryo-EM structure PDB 9NYR [66, 67]. Given that the two experimental structures exhibit substantial structural divergence (Cα RMSD ~10 Å), these findings underscore the predictive strength of our approach and suggest that multiple productive conformations per system could be experimentally confirmed. On the other hand, modeled conformations in which POI lysines were positioned >50 Å from E2 Cys111 exhibited high stability and showed no conformational rearrangements capable of repositioning these residues into the ubiquitination range. This finding aligns with the possibility that rigid POI–E3 ligase conformations can arise in which solvent-accessible lysine residues are not positioned for ubiquitination, resulting in unsuccessful POI degradation [18].

After analyzing the experimental benchmark dataset, we applied our computational workflow to model WEE1-CRBN and PKMYT1-CRBN PROTACs, selected to assess workflow generalizability in the absence of experimental ternary complex structures. Seven WEE1-CRBN and six PKMYT1-CRBN representative conformations exhibiting feasible warhead connectivity and sustained ubiquitination accessibility were identified. These conformations span a diverse set of geometrically plausible assemblies suitable for further evaluation of linker design and assessment of PROTAC-induced degradation potential. Docking energies of representative conformations were independent of conformational sampling populations or ubiquitinability, indicating that POI–E3 binding affinity is inadequate to predict degradation outcome and that multiple conformations must be considered in modeling efforts. However, induced-fit docking of active PROTACs showed that, despite conformational diversity among the WEE1-CRBN models, all conformations were capable of accommodating four of the six WEE1 PROTACs examined, whereas the remaining two PROTACs were exclusively accommodated either in a single model (WEE1-CRBN–M5 in the case of ZNL-02-096) or in two models (WEE1-CRBN–M5 and M8 in the case of TL12-186). In this context, PROTAC docking scores were used to prioritize the generated PROTAC ternary complexes for further evaluation, while accounting for the possibility that the same PROTAC may engage distinct productive POI–E3 conformations. Induced-fit docking of the PKMYT1 PROTAC D16-M1P2 was successful only in PKMYT1-CRBN–M4 irrespective of linker stereochemistry, consistent with the developers’ claim that variations in linker stereochemistry do not impact PROTAC activity [50]. Analysis of linker conformations revealed a strong correlation between PROTAC activity and linker compatibility with attachment-atom distance windows derived from productive POI–E3 models. Active WEE1 and PKMYT1 PROTACs adopted linker lengths matching these windows, whereas linkers of inactive PKMYT1 PROTACs were too short to bridge productive PKMYT1-CRBN geometries. These results highlight that appropriate linker length is critical for ternary complex formation and provide a rational framework for designing linkers with optimal degradation potential. Recently, Kumar and Sophia addressed the principle that degradation competence depends on ubiquitination accessibility, conformational diversity, and linker-dependent geometry by employing an integrative computational framework with an application to PTP1B-VHL system [68]. Our work extends this approach by introducing quantitative, transferable design rules with explicit numerical thresholds and by validating generalizability across additional POI–E3 ligase systems (WEE1- and PKMYT1-CRBN). Together, these modeling efforts provide a more comprehensive understanding of target protein degradation beyond static experimental snapshots and offer mechanistically grounded strategies for future degrader design.

## Conclusion

This study demonstrates that productive degradation arises from a balance of conformational diversity and preserved ubiquitination competence, rather than from ternary complex stability or binding affinity alone. By integrating structural benchmarking, connectivity constraints, conformational clustering, ubiquitination accessibility metrics and MD simulations, we establish a generalizable computational framework for identifying productive POI–E3 ligase models. Quantitative thresholds for warhead attachment distances, Cα RMSD-based conformational divergence and lysine proximity to the E2 catalytic site provide mechanistically interpretable design rules that can be applied prospectively. Application to WEE1-CRBN and PKMYT1-CRBN systems reveals that multiple productive conformations must be considered during PROTAC design and that linker length compatibility with the attachment atom distance windows is a critical determinant of activity. Importantly, the decoupling of POI–E3 binding affinity from degradation potential highlights the need to move beyond static or single-structure paradigms. Future impact lies in accelerating this workflow (e.g., through fast AI-assisted conformational sampling beyond the limited set of dominant cofolding predictions), incorporating enhanced sampling strategies to identify additional ubiquitination-competent conformations and in extending it to additional degraders and E3 ligases. Together, these advances will enable more predictive, mechanism-driven design of next-generation targeted protein degraders.

## Supplementary Information

Additional file1 (ZIP 4652 KB)

Additional file2 (DOCX 34073 KB)

## Data Availability

All scripts and data required to reproduce the results of this study are publicly available at: https://github.com/Husam-PSE/PROTACMap. The repository includes example input files, processed outputs and a detailed description of the computational workflow. Software dependencies and versions are specified to ensure reproducibility.
