# Supplementary material for: Computational mapping of productive POI–E3 ligase conformations to guide de novo degrader design: application to WEE1 and PKMYT1 PROTACs
Source: J Cheminform. 2026 Jul 24;18:103. doi: 10.1186/s13321-026-01268-5 (PMC13404913; doi:10.1186/s13321-026-01268-5)
Supplement: Supplementary file 2 — Additional file2 (DOCX 34073 KB) [file 13321_2026_1268_MOESM2_ESM.docx]

**Supplementary Information for:**

**Computational Mapping of Productive POI–E3 Ligase Conformations to Guide De Novo Degrader Design: Application to WEE1 and PKMYT1 PROTACs**

Husam Nassar^1^, Matthias Schmidt^1^, Hany S. Ibrahim^1^, Dina Robaa^1^, Wolfgang Sippl^1*^

^1^Department of Medicinal Chemistry, Institute of Pharmacy, Martin-Luther University Halle-Wittenberg, Halle (Saale), Germany.

^*^Corresponding author: Wolfgang Sippl

Email: [wolfgang.sippl@pharmazie.uni-halle.de](mailto:wolfgang.sippl@pharmazie.uni-halle.de)

Table of Contents

1. **Structural Analysis of Experimental PROTAC Ternary Complexes**3
   1. Static Analysis of PROTAC Linker Lengths3
   2. Inspection of Static POI-E3 Ligase Conformations4
   3. Ubiquitinability Assessment of Static POI-E3 Ligase Conformations5
   4. Cα RMSD over 1 µs MD Simulations10
   5. Linker Length Distributions over 1 µs MD Simulations11
   6. Ubiquitination Accessibility over 1 µs MD Simulations12
2. **Modeling of Experimental PROTAC Ternary Structures**13
   1. CRBN-mediated Systems13
      1. BRD4BD1-CRBN13
      2. BRD4BD2-CRBN16
      3. PTPN2-CRBN19
      4. CDK2-CRBN22
   2. VHL-mediated Systems25
      1. SMARCA2-VHL25
      2. SMARCA4-VHL28
      3. BclxL-VHL31
      4. BCL2-VHL34
      5. WDR5-VHL37
      6. BRD4BD1-VHL40
      7. BRD4BD2-VHL43
      8. KRas-VHL46
      9. FAK-VHL49
      10. WEE1-VHL52
3. **POI-E3 Conformational Sampling Convergence Analysis**55
   1. Cluster Saturation Analysis55
   2. Cluster Population Analysis56
4. **Modeling of WEE1 and PKMYT1 CRBN-mediated PROTACs**57
   1. Pharmacophore Features Guiding PROTAC Induced-fit Docking57
   2. PROTAC-Protein Interaction Occupancy Rates over 1 µs MD Simulations58
      1. MA071, ZNL-02-012, ZNL-02-040 and ZNL-02-047 in WEE1-CRBN–M358
      2. ZNL-02-096 and TL12-186 in WEE1-CRBN–M559
      3. D16-M1P2 in PKMYT1-CRBN–M460
   3. Ubiquitination Accessibility of Modelled ternary complexes61
   4. Attachment Atom Distances to Guide Linker Design64
5. **Synthesis of PROTACs HI100-103**67
6. **Analytical Charts of PROTACs HI100-103**75
7. **References**81
8. **Structural Analysis of Experimental PROTAC Ternary Complexes**
   1. Static Analysis of PROTAC Linker Lengths

**Table S1:** Summary of PROTAC linker lengths, corresponding POIs and ternary complex PDB IDs.

| **E3 Ligase** | **POI** | **PROTAC ID** | **Linker Length (Å)** | **Ternary Complex PDB ID** |
| --- | --- | --- | --- | --- |
| **CRBN** | BRD4BD1 | dBET23 | 9.73 | 6BN7 |
|  |  | dBET6 | 13.24 | 6BOY |
|  | BRD4BD2 | CFT-1297 | 13.49 | 8RQ9 |
|  | PTPN2 | PROTAC1 | 5.47 | 8UH6 |
|  | CDK2 | Compound 4 | 12.76 | 9D0W |
|  |  | Compound 24 | 10.60 | 9NYR |
| **VHL** | SMARCA2 | PROTAC2 | 7.93 | 6HAX |
|  |  | PROTAC1 | 8.33 | 6HAY |
|  |  | ABCI1 | 9.84 | 7S4E |
|  |  | PROTAC5 | 5.00 | 7Z6L |
|  |  | PROTAC10 | 4.80 | 7Z76 |
|  |  | PROTAC6 | 4.47 | 7Z77 |
|  | SMARCA4 | PROTAC1 | 4.84 | 8G1Q |
|  | Bcl-xL | PROTAC6 | 12.55 | 6ZHC |
|  |  | 735b | 11.02 | 8FY0 |
|  | BCL2 | 753b | 12.59 | 8FY1 |
|  |  | WH244 | 11.00 | 8FY2 |
|  | WDR5 | PROTAC MS33 | 11.92 | 7JTO |
|  |  | PROTAC MS67 | 4.73 | 7JTP |
|  |  | PROTAC PEG2 | 13.76 | 8BB2 |
|  |  | PROTAC PEG1 | 11.59 | 8BB3 |
|  |  | AD157 | 6.87 | 8BB4 |
|  |  | AD122 | 9.06 | 8BB5 |
|  | BRD4BD1 | PROTAC9 | 8.26 | 7KHH |
|  |  | PROTAC48 | 8.94 | 8BDS |
|  |  | PROTAC49 | 8.49 | 8BEB |
|  | BRD4BD2 | MZ1 | 9.25 | 5T35 |
|  |  | AT7 | 7.75 | 7ZNT |
|  |  | PROTAC51 | 7.61 | 8BDT |
|  |  | PROTAC48 | 7.88 | 8BDX |
|  | KRas | PROTAC3 | 7.51 | 8QW6 |
|  |  | PROTAC4 | 8.60 | 8QW7 |
|  | FAK | GSK215 | 2.49 | 7PI4 |
|  | WEE1 | AZD1775 | 6.60 | 8WDK |

- 1. Inspection of Static POI-E3 Ligase Conformations

| 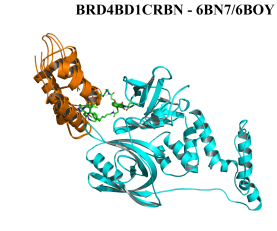 | 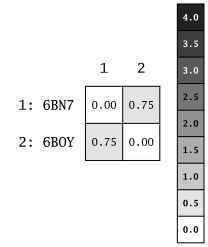 | 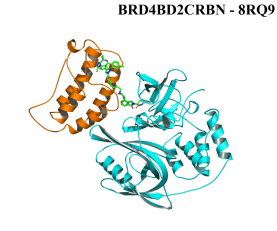 | | 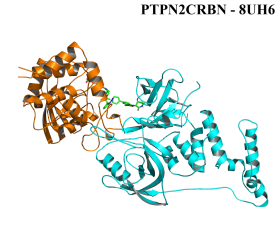 | | 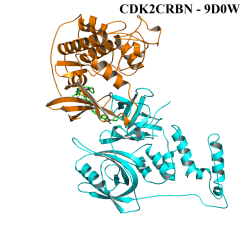 |
| --- | --- | --- | --- | --- | --- | --- |
| 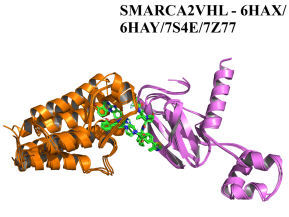 | 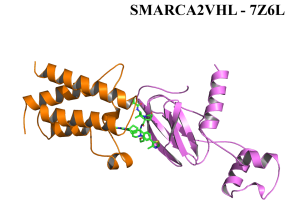 | 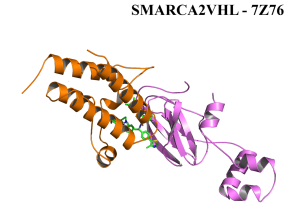 | | 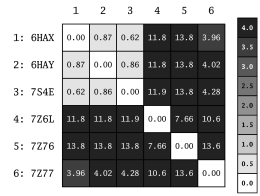 | | 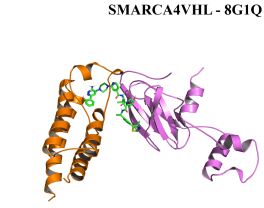 |
| 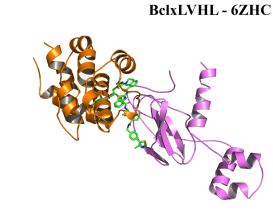 | 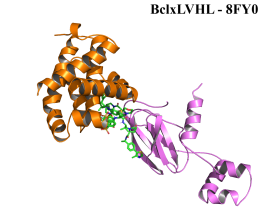 | 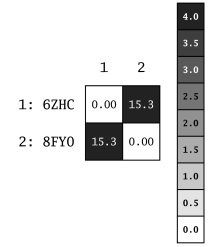 | | 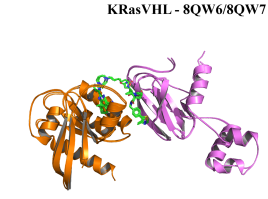 | | 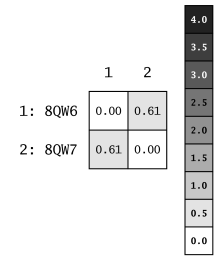 |
| 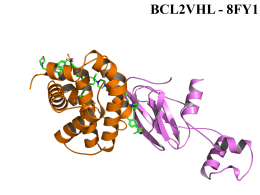 | 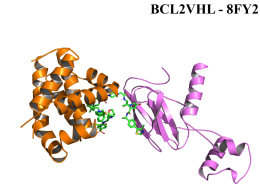 | 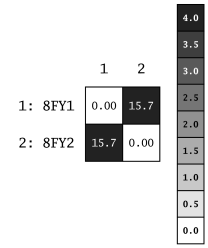 | | 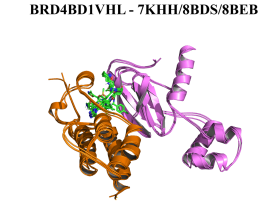 | 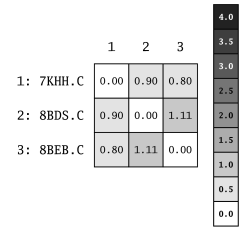 | |
| 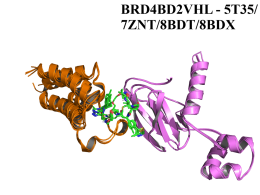 | 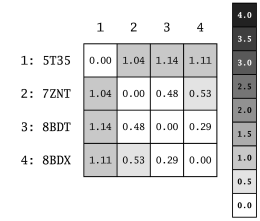 | 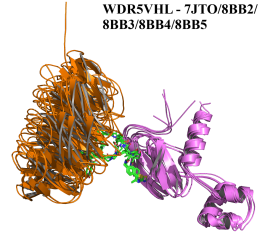 | | 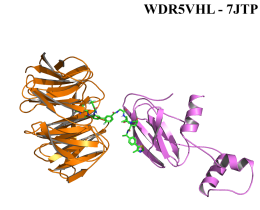 | | 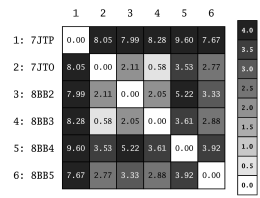 |
| 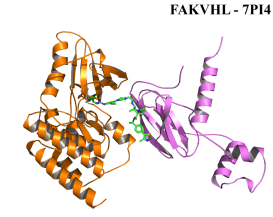 | | | 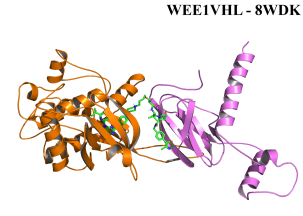 | | | |
| **Figure S1:** Cartoon representations of experimental POI–E3 ligase conformations with corresponding pairwise Cα RMSD values. | | | | | | |

- 1. Ubiquitinability Assessment of Static POI-E3 Ligase Conformations

| 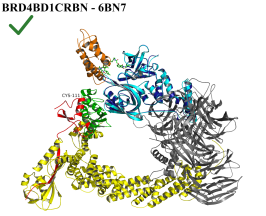 | 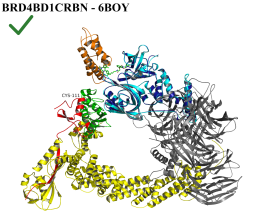 | 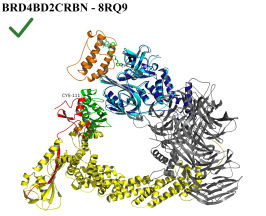 | 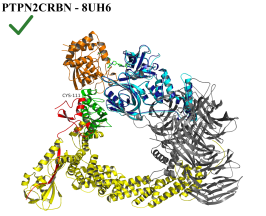 | 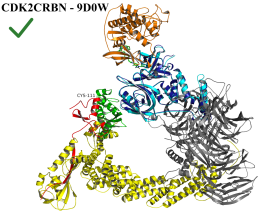 |
| --- | --- | --- | --- | --- |
| 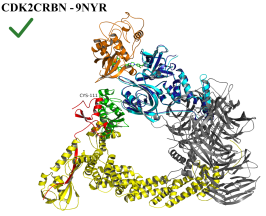 | 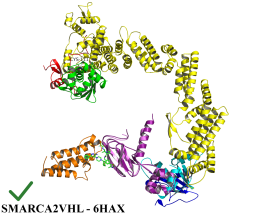 | 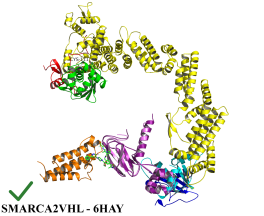 | 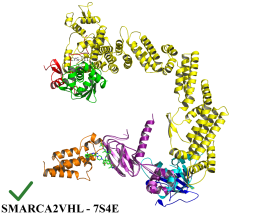 | 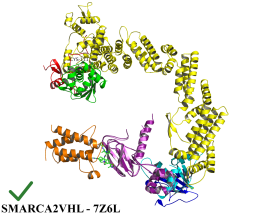 |
| 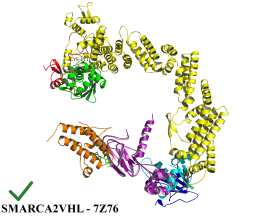 | 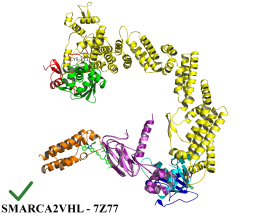 | 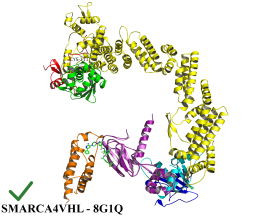 | 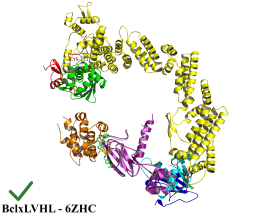 | 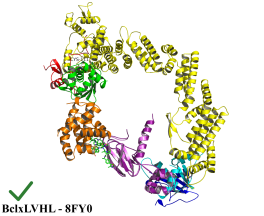 |
| 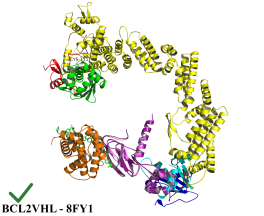 | 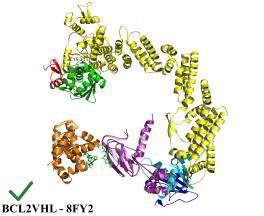 | 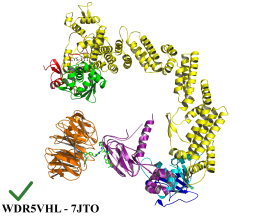 | 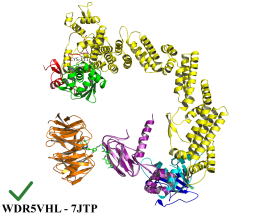 | 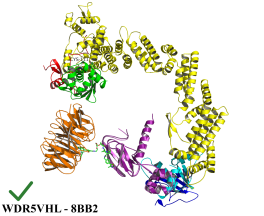 |
| 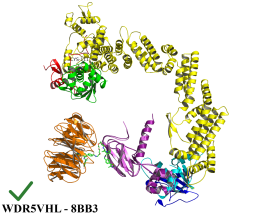 | 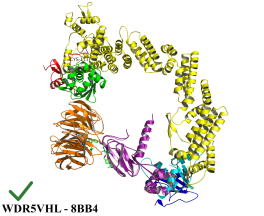 | 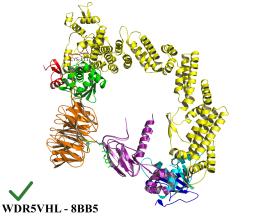 | 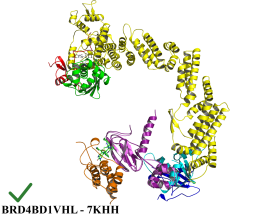 | 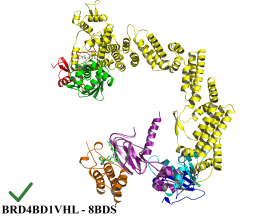 |
| 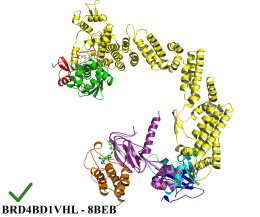 | 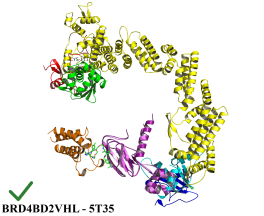 | 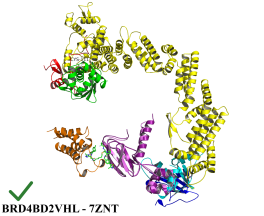 | 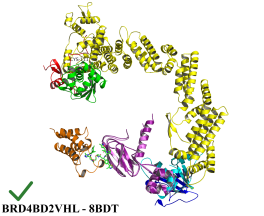 | 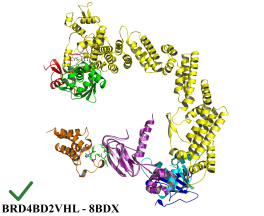 |
| 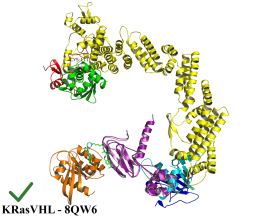 | 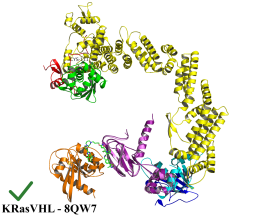 | 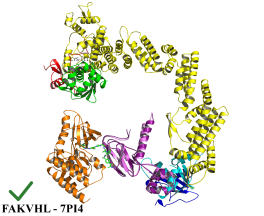 | 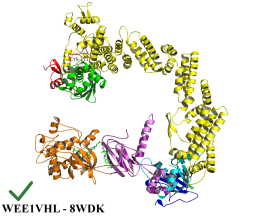 |  |
| **Figure S2:** Experimental POI-E3 ligase conformations incorporated into the corresponding CRL4CRBN or CRL2VHL modeled ubiquitination assemblies. | | | | |

| **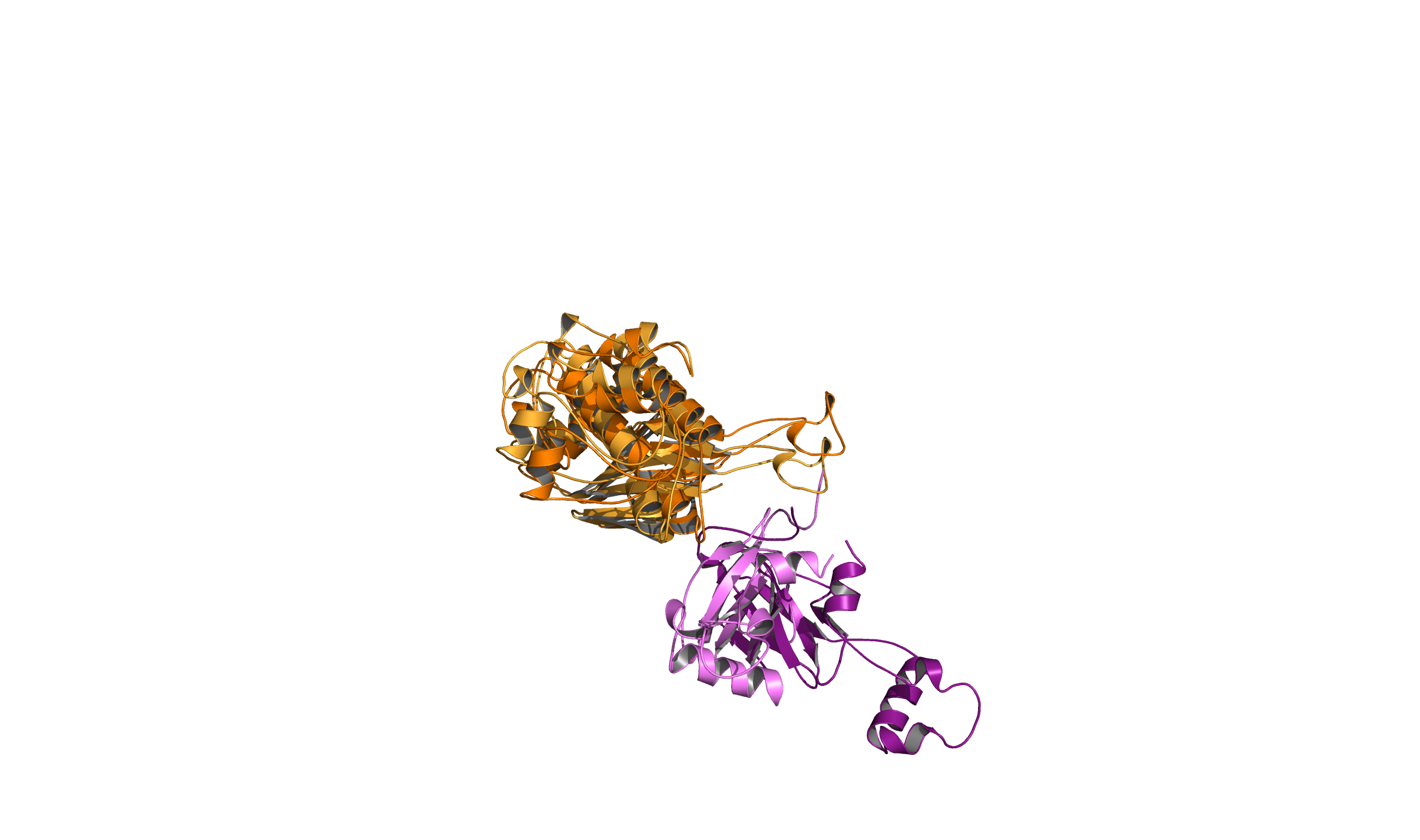**  WEE1-VHL models X and Y | **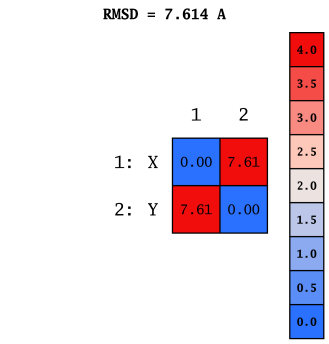** | **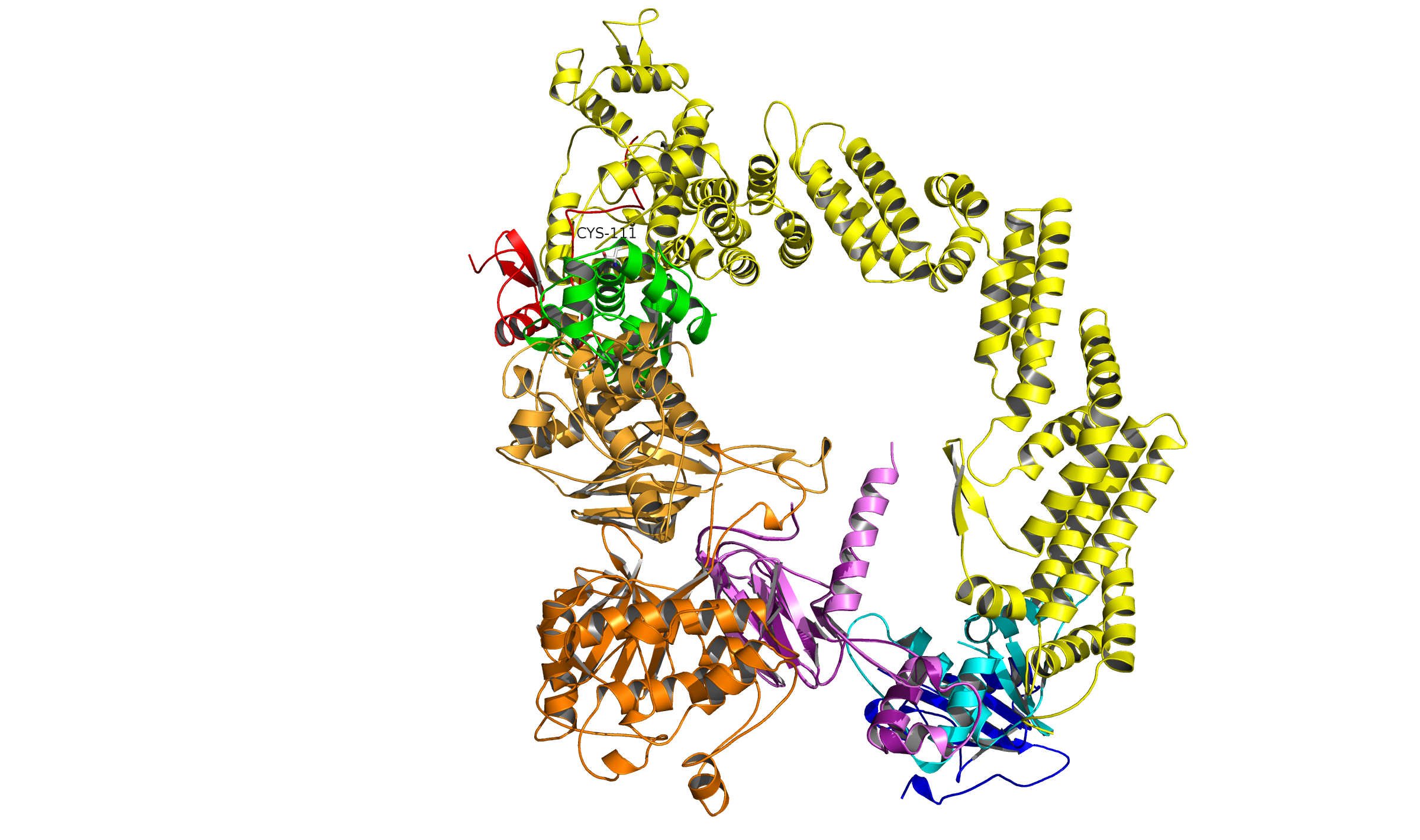**  WEE1-VHL model X is clashing with E2 subunit. Model Y is inaccessible to ubiquitination. Superposition of the two models showed Cα RMSD > 7.5 Å | |
| --- | --- | --- | --- |
| **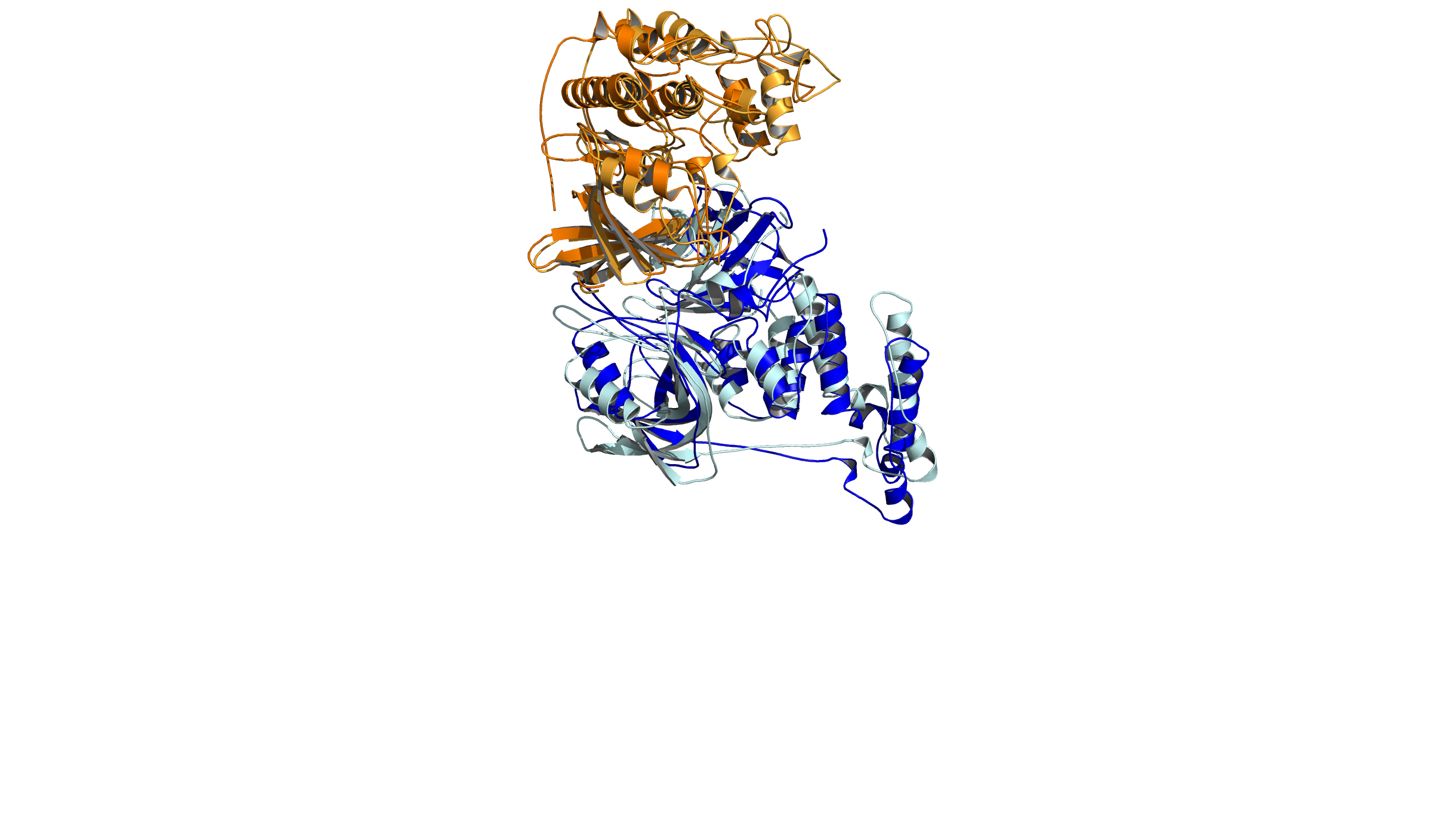**  CDK2-CRBN models X and Y | **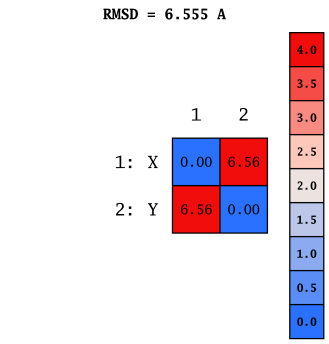** | **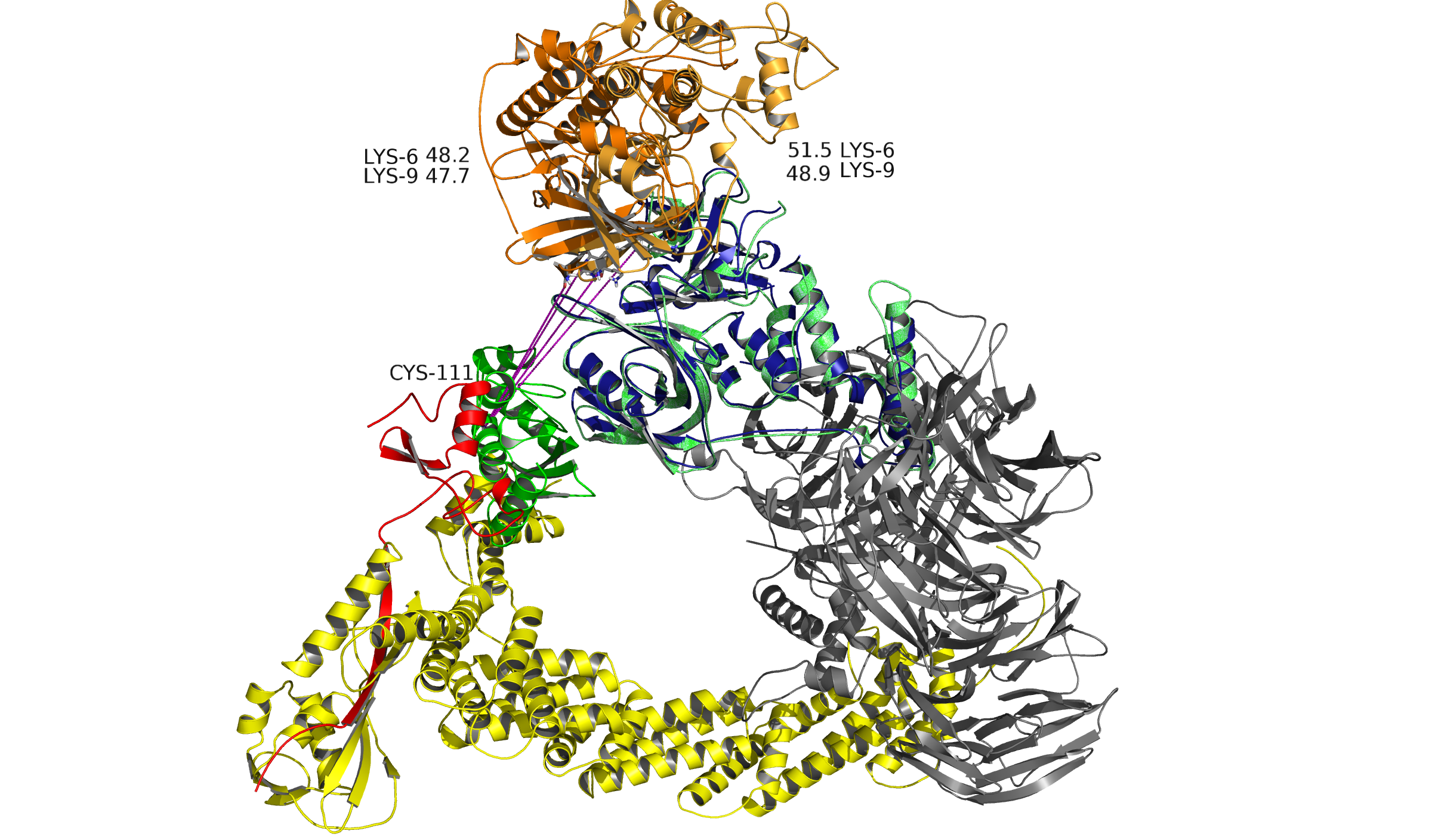**  CDK2-CRBN models X and Y showing similar ubiquitination accessibility patterns. Superposition of the two models showed Cα RMSD < 7.5 Å | |
| **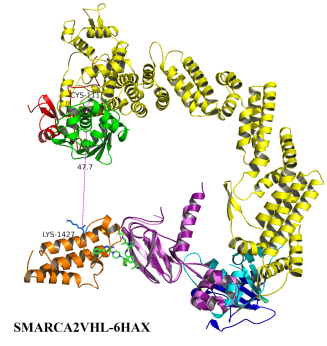** | **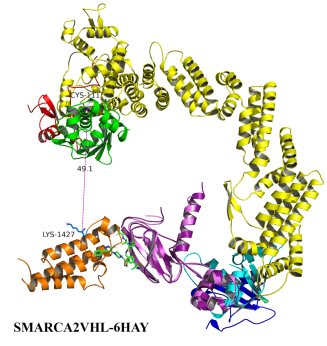** | **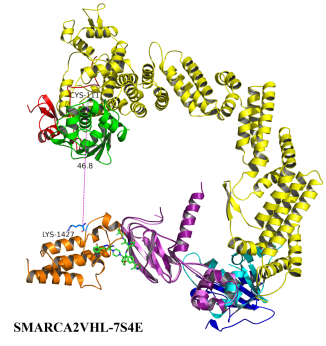** | **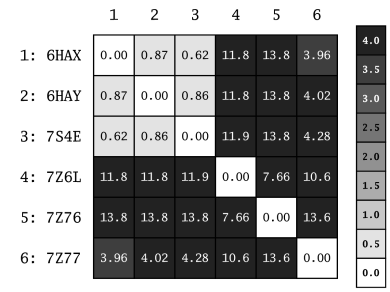** |
| **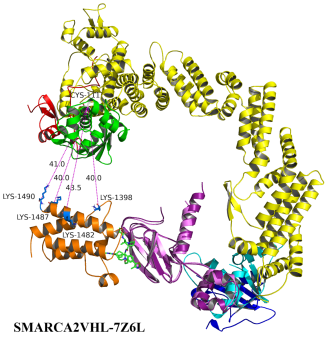** | **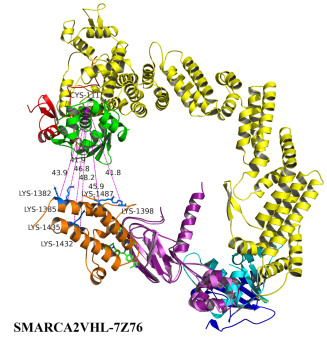** | **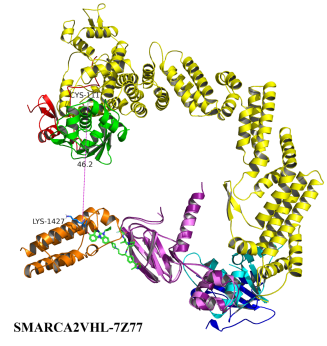** |  |
| **Figure S3:** Observations during modeling, demonstrating that conformations differing by more than 7.5 Å typically exhibited different ubiquitination competence profiles. SMARCA2 ubiquitination accessibility profiles compared with pairwise Cα RMSD analyses are shown in the third row, highlighting correlations between ubiquitination sites and SMARCA2-VHL conformational divergence. | | | |

| 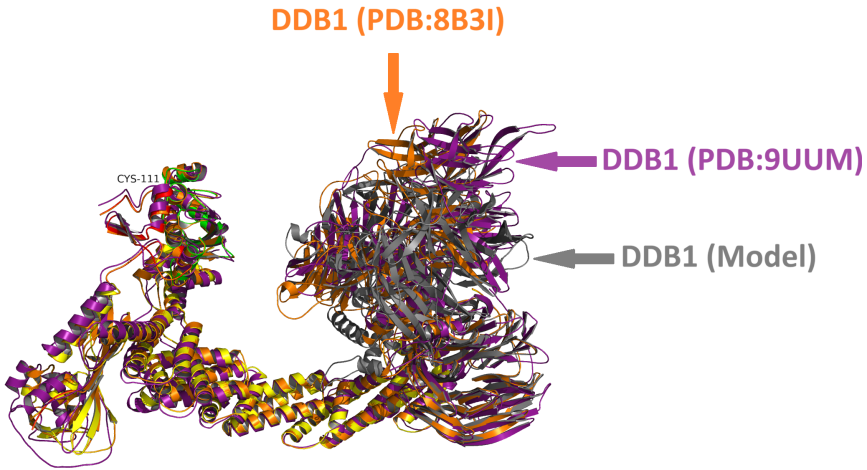 | | | | | | | |
| --- | --- | --- | --- | --- | --- | --- | --- |
| 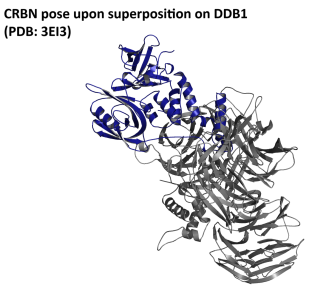 | | 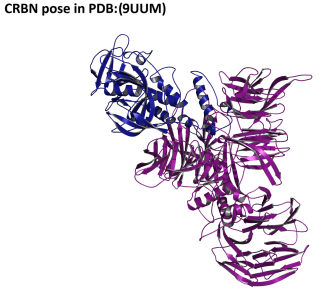 | | | 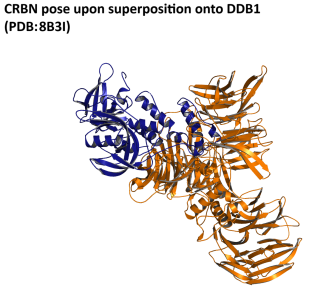 | | |
| 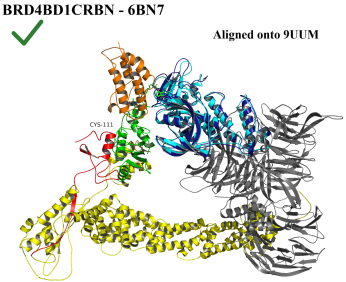 | 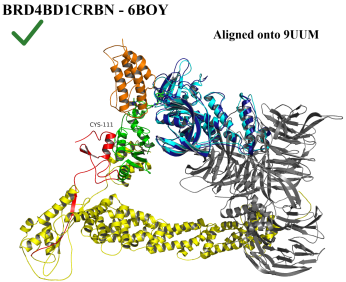 | | | 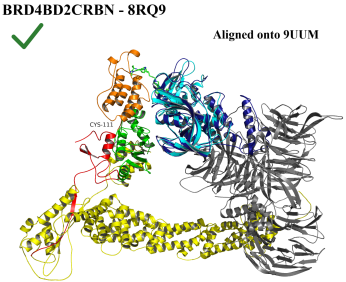 | | | 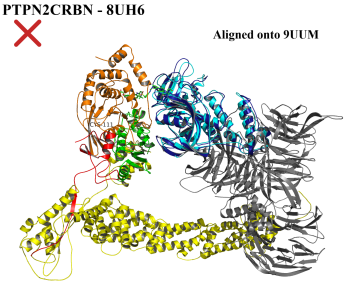 |
| 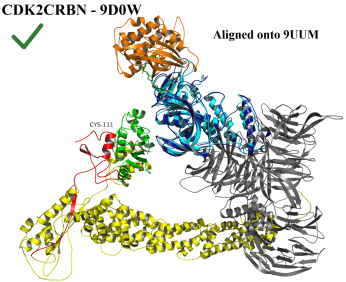 | | | 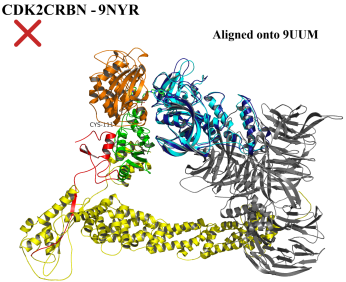 | | | 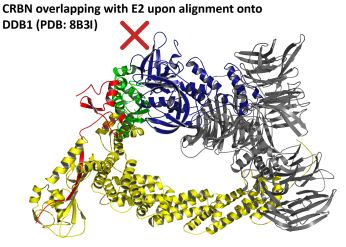 | |
| **Figure S4:** Comparison of CRBN and POI positioning within different CRL4CRBN assemblies | | | | | | | |

| 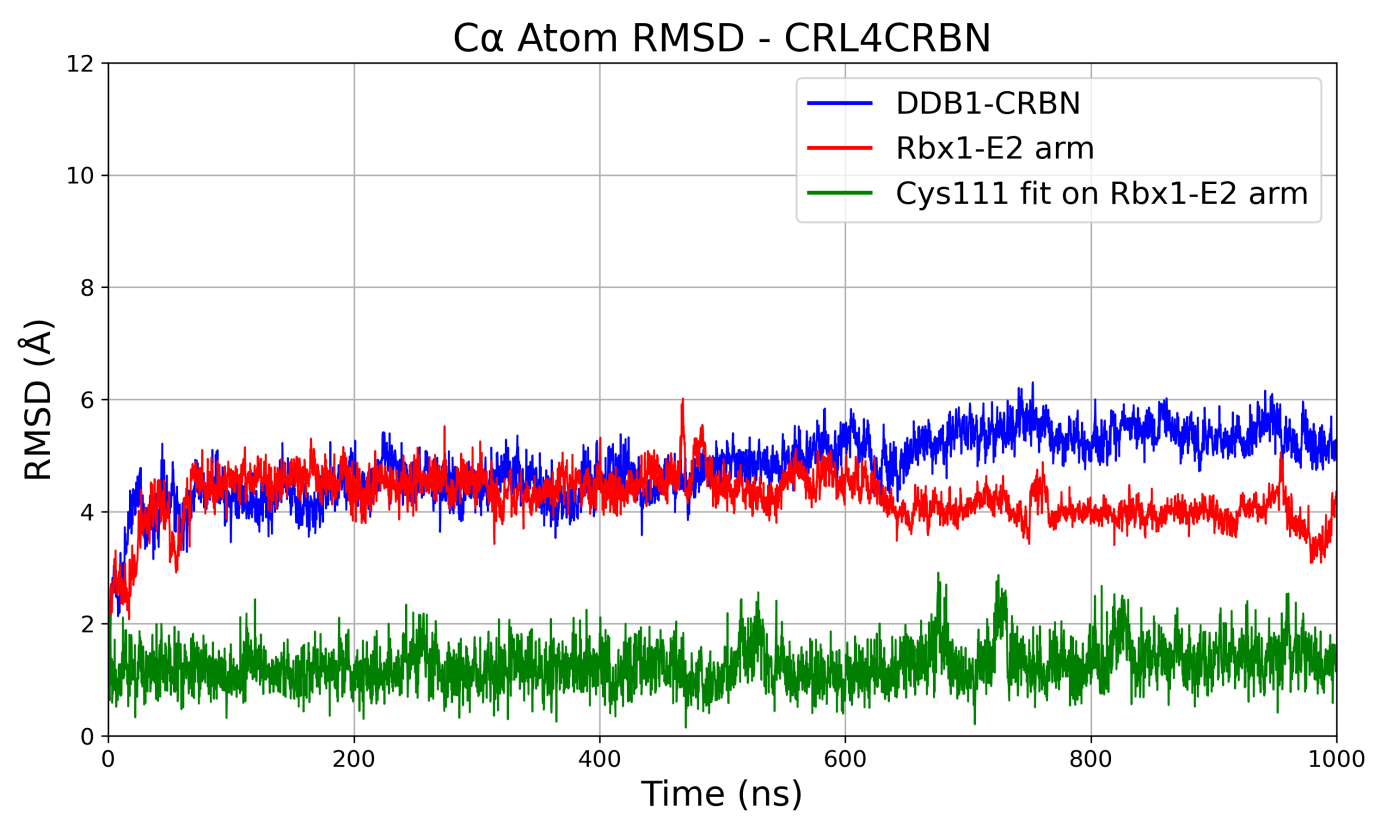 |
| --- |
| 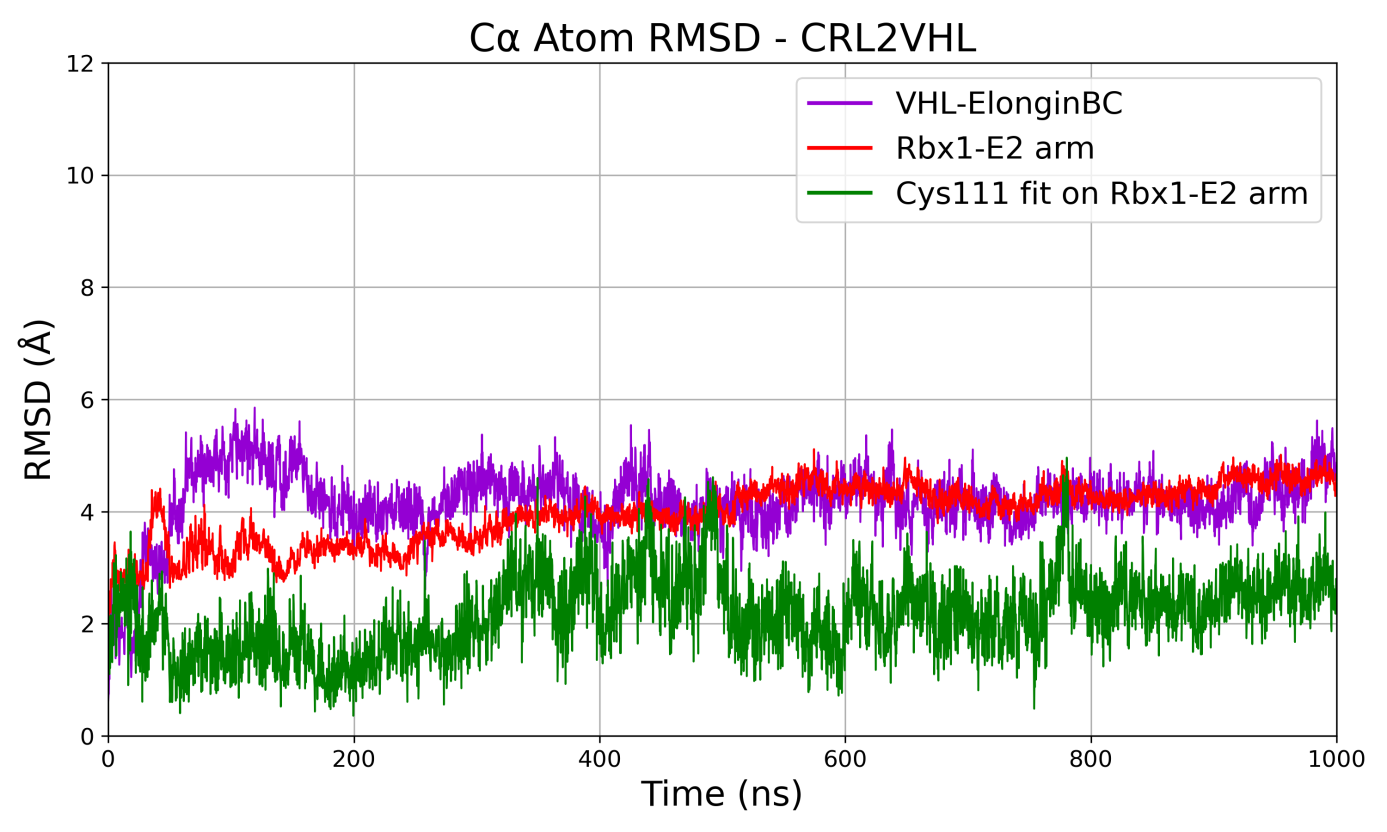 |
| **Figure S5:** Cα RMSD values of CRBN-DDB1 and VHL-Elongin BC complexes as well as Rbx1-E2 arm of the modeled ubiquitination assemblies over 1 µs MD simulations. |

**Table S2:** Ubiquitinable solvent-exposed POI lysine residues.

| **PROTAC Ternary Complex (PDB)** | **Ubiquitinable POI Lysine Residues (Static analysis) Solvent Exposed Lysine Residues within 50Å of E2 Cys111** |
| --- | --- |
| BRD4BD1-dBET23-CRBN (6BN7) | K72/K76/K91/K99/K102/K111/K112/K141/K155/K160 |
| BRD4BD1-dBET6-CRBN (6BOY) | K72/K76/K91/K99/K102/K111/K112/K141/K155/K160 |
| BRD4BD2-CFT1297-CRBN (8RQ9) | K346/K349/K355/K362/K378/K395/K404/K431/K445/K456 |
| PTPN2-PROTAC1-CRBN (8UH6) | K60/K105/K107/K118/K137/K144/K151/K198/K238/K253/K277 |
| CDK2-Compound4-CRBN (9D0W) | K6/K9/K89 |
| CDK2-Compound24-CRBN (9NYR) | K6/ K9/ K24/ K34/ K56/ K65/ K88/ K89/ K278/K291 |
| SMARCA2-PROTAC2-VHL (6HAX) | K1427 |
| SMARCA2-PROTAC1-VHL (6HAY) | K1427 |
| SMARCA2-ABCI1-VHL (7S4E) | K1427 |
| SMARCA2-PROTAC5-VHL (7Z6L) | K1398/K1482/K1487/K1490 |
| SMARCA2-PROTAC10-VHL (7Z76) | K1382/K1385/K1398/K1432/K1435/K1487 |
| SMARCA2-PROTAC6-VHL (7Z77) | K1427 |
| SMARCA4-PROTAC1-VHL (8G1Q) | K1492/K1503 |
| BclxL-PROTAC6-VHL (6ZHC) | K87 |
| BclxL-735b-VHL (8FY0) | K16/K20/K87/K157 |
| BCL2-735b-VHL (8FY1) | K22 |
| BCL2-WH244-VHL (8FY2) | K17/K22 |
| WDR5-MS33-VHL (7JTO) | K207/K227/K245/K247/K256/K259/K272/K291/K296 |
| WDR5-MS67-VHL (7JTP) | K32/K38/K46/K67/K78/K256/K259/K272/K291/K296/K325/K331 |
| WDR5-PEG2-VHL (8BB2) | K207/K227/K245/K247/K256/K259/K272/K291/K296/K331 |
| WDR5-PEG1-VHL (8BB3) | K207/K227/K245/K247/K256/K259/K272/K291/K296 |
| WDR5-AD157-VHL (8BB4) | K165/ K207/K227/K245/K247/K256/K259/K272/K291/K296 |
| WDR5-AD122-VHL (8BB5) | K207/K227/K245/K247/K256/K259/K272/K291/K296/K331 |
| BRD4BD1-PROTAC9-VHL (7KHH) | K91 |
| BRD4BD1-PROTAC48-VHL (8BDS) | K91 |
| BRD4BD1-PROTAC49-VHL (8BEB) | K91 |
| BRD4BD2-MZ1-VHL (5T35) | K362/K445/K456 |
| BRD4BD2-AT7-VHL (7ZNT) | K362/K445/K456 |
| BRD4BD2-PROTAC51-VHL (8BDT) | K362/K445/K456 |
| BRD4BD2-PROTAC48-VHL (8BDX) | K362/K445/K456 |
| KRas-PROTAC3-VHL (8QW6) | K88 |
| KRas-PROTAC4-VHL (8QW7) | K88 |
| FAK-GSK215-VHL (7PI4) | K457/K467/K485/K583 |
| WEE1-AZD1775-VHL (8WDK) | K399/K547 |

- 1. Cα RMSD over 1 µs MD Simulations

| 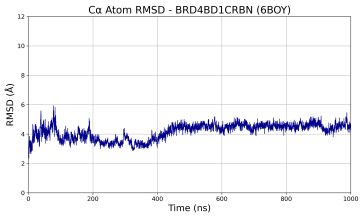 | 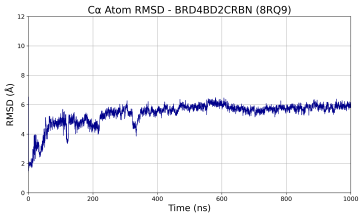 | | 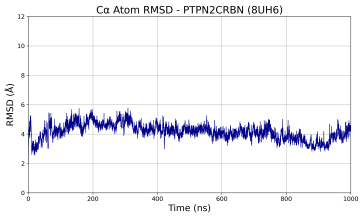 | | 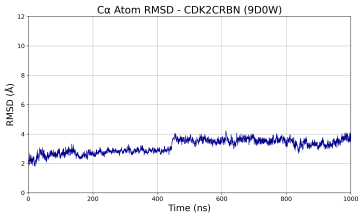 |
| --- | --- | --- | --- | --- | --- |
| 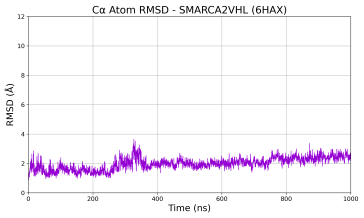 | | 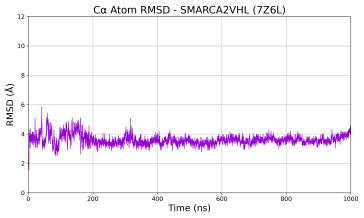 | | 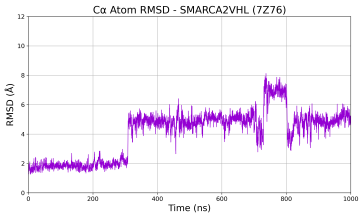 | |
| 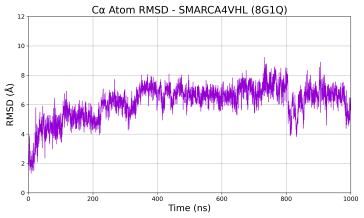 | | 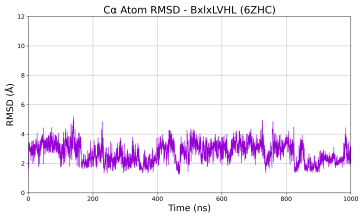 | | 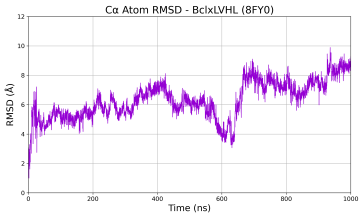 | |
| 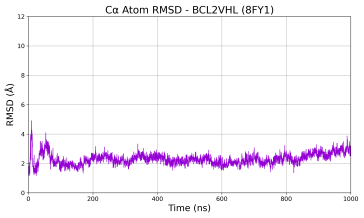 | | 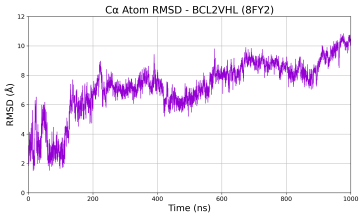 | | 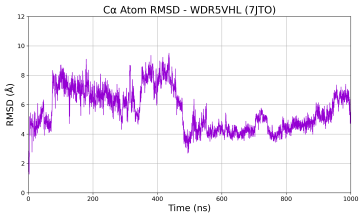 | |
|  | |  | |  | |
|  | |  | |  | |
| **Figure S6:** Cα RMSD values of experimental ternary complexes over 1 µs MD simulations. | | | | | |

- 1. Linker Length Distributions over 1 µs MD Simulations

|  |  | |  | |  |
| --- | --- | --- | --- | --- | --- |
|  | |  | |  | |
|  | |  | |  | |
|  | |  | |  | |
|  | |  | |  | |
|  | |  | |  | |
| **Figure S7:** Linker lengths of experimental PROTAC structures over 1 µs MD simulations. | | | | | |

- 1. Ubiquitination Accessibility over 1 µs MD Simulations

|  |  | |  | |  |
| --- | --- | --- | --- | --- | --- |
|  | |  | |  | |
|  | |  | |  | |
|  | |  | |  | |
|  | |  | |  | |
|  | |  | |  | |
| **Figure S8:** Ubiquitination accessibility heatmaps of POI lysine residues over 1 µs MD Simulations. | | | | | |

1. **Modeling of Experimental PROTAC Ternary Structures**
   1. CRBN-mediated systems
      1. BRD4BD1-CRBN

|  | |  | |
| --- | --- | --- | --- |
|  | |  | |
|  |  |  |  |
|  |  |  |  |
| **Figure S9:** Docking, filtering, clustering and ubiquitination accessibility of BRD4BD1-CRBN conformations. | | | |

**Table S3:** Clustering statistics and docking energy scores of representative BRD4BD1-CRBN conformations.

| **Cluster ID** | **Size** | **Average Pairwise RMSD** | **Representative BRD4BD1CRBN Conformation** | **Representative S Docking Score (Kcal/mol)** |
| --- | --- | --- | --- | --- |
| 3 | 22 | 5.73 | M6_P-P273 | -34.88 |
| 2 | 15 | 5.66 | M1_P-P23 | -53.73 |
| 5 | 9 | 4.72 | M4_P-P150 | -40.52 |
| 1 | 7 | 4.32 | M2_P-P52 | -48.40 |
| 4 | 3 | 1.88 | M7_P-P282 | -34.49 |
| 6 | 1 | 0.00 | M3_P-P129 | -41.64 |
| 7 | 1 | 0.00 | M5_P-P153 | -40.31 |

| - **Cα RMSD of M6_P-P273 fitting on:** - **PDB Structure 6BN7 = 4.60 Å** - **PDB Structure 6BOY = 4.85 Å** |
| --- |

|  |  |  |
| --- | --- | --- |
|  |  |  |
|  |  |  |
|  |  |  |
|  |  |  |
| **Figure S10:** Cα RMSD, attachment atom distances and ubiquitination accessibility of BRD4BD1-CRBN conformations over 100 ns MD simulations. | | |

- - 1. BRD4BD2-CRBN

|  |  | |
| --- | --- | --- |
|  |  | |
|  |  |  |
|  |  |  |
| **Figure S11:** Docking, filtering, clustering and ubiquitination accessibility of BRD4BD2-CRBN conformations. | | |

**Table S4:** Clustering statistics and docking energy scores of representative BRD4BD2-CRBN conformations.

| **Cluster ID** | **Size** | **Average Pairwise RMSD** | **Representative BRD4BD2CRBN Conformation** | **Representative S Docking Score (Kcal/mol)** |
| --- | --- | --- | --- | --- |
| 4 | 97 | 5.51 | M1_P-P2 | -65.05 |
| 3 | 15 | 4.16 | M2_P-P113 | -41.95 |
| 2 | 6 | 1.76 | M4_P-P290 | -31.57 |
| 1 | 3 | 2.15 | M3_P-P122 | -41.58 |

| - **Cα RMSD of M1_P-P2 fitting on:** - **PDB Structure 8RQ9 = 3.60 Å** |
| --- |

|  |  |  |
| --- | --- | --- |
|  |  |  |
|  |  |  |
|  |  |  |
| **Figure S12:** Cα RMSD, attachment atom distances and ubiquitination accessibility of BRD4BD2-CRBN conformations over 100 ns MD simulations. The red X indicates that the model was excluded from the attachment-atom distance monitoring due to its ubiquitination inaccessibility. | | |

- - 1. PTPN2-CRBN

|  | | |  | | |
| --- | --- | --- | --- | --- | --- |
|  | | |  | | |
|  | |  | |  |  |
|  |  |  | |  |  |
|  |  |  | |  |  |
|  |  |  | |  |  |
| **Figure S13:** Docking, filtering, clustering and ubiquitination accessibility of PTPN2-CRBN conformations. | | | | | |

**Table S5:** Clustering statistics and docking energy scores of representative PTPN2-CRBN conformations.

| **Cluster ID** | **Size** | **Average Pairwise RMSD** | **Representative  PTPN2CRBN Conformation** | **Representative S Docking Score (Kcal/mol)** |
| --- | --- | --- | --- | --- |
| 10 | 41 | 5.01 | M2_P-P25 | -59.77 |
| 7 | 29 | 5.43 | M1_P-P16 | -62.16 |
| 11 | 19 | 4.84 | M12_P-P241 | -40.12 |
| 13 | 12 | 4.92 | M13_P-P336 | -36.17 |
| 5 | 9 | 3.50 | M9_P-P143 | -45.26 |
| 1 | 6 | 2.17 | M3_P-P42 | -54.59 |
| 3 | 3 | 6.12 | M6_P-P112 | -46.63 |
| 4 | 3 | 2.52 | M4_P-P66 | -51.58 |
| 8 | 3 | 5.70 | M10_P-P166 | -43.36 |
| 9 | 3 | 1.87 | M5_P-P82 | -49.07 |
| 12 | 3 | 2.74 | M8_P-P134 | -45.66 |
| 2 | 2 | 3.23 | M7_P-P123 | -46.15 |
| 6 | 2 | 5.28 | M11_P-P212 | -41.03 |

| - **Cα RMSD of M4_P-P66 fitting on:** - **PDB Structure 8UH6 = 4.90 Å** |
| --- |

|  |  |  |
| --- | --- | --- |
|  |  |  |
|  |  |  |
|  |  |  |
|  |  |  |
|  |  |  |
|  |  |  |
|  |  |  |
|  |  |  |
|  |  |  |
| **Figure S14:** Cα RMSD, attachment atom distances and ubiquitination accessibility of PTPN2-CRBN conformations over 100 ns MD simulations. | | |

- - 1. CDK2-CRBN

|  | |  | |
| --- | --- | --- | --- |
|  | |  | |
|  |  |  |  |
|  |  |  |  |
|  |  |  |  |
| **Figure S15:** Docking, filtering, clustering and ubiquitination accessibility of CDK2-CRBN conformations. | | | |

**Table S6:** Clustering statistics and docking energy scores of representative CDK2-CRBN conformations.

| **Cluster ID** | **Size** | **Average Pairwise RMSD** | **Representative BRD4BD2CRBN Conformation** | **Representative S Docking Score (Kcal/mol)** |
| --- | --- | --- | --- | --- |
| 9 | 40 | 5.10 | M5_P-P98 | -51.26 |
| 4 | 8 | 3.27 | M4_P-P44 | -56.69 |
| 5 | 6 | 4.96 | M8_P-P432 | -38.12 |
| 7 | 6 | 3.88 | M7_P-P197 | -45.95 |
| 1 | 5 | 3.61 | M3_P-P42 | -57.05 |
| 3 | 5 | 3.75 | M2_P-P13 | -65.19 |
| 2 | 4 | 2.40 | M1_P-P3 | -78.32 |
| 8 | 3 | 3.44 | M6_P-P103 | -50.86 |
| 6 | 1 | 0.00 | M9_P-P819 | -25.42 |

| - **Cα RMSD of M7_P-P197 fitting on:** - **PDB Structure 9D0W = 4.80 Å** | - **Cα RMSD of M6_P-P103 fitting on:** - **PDB Structure 9NYR = 5.00 Å** |
| --- | --- |

|  |  |  |
| --- | --- | --- |
|  |  |  |
|  |  |  |
|  |  |  |
|  |  |  |
| **Figure S16:** Cα RMSD, attachment atom distances and ubiquitination accessibility of CDK2-CRBN conformations over 100 ns MD simulations. | | |

- 1. **VHL-mediated Systems**
     1. SMARCA2-VHL

|  | |  | |
| --- | --- | --- | --- |
|  | |  | |
|  | |  |  |
|  |  |  |  |
|  |  |  |  |
| **Figure S17:** Docking, filtering, clustering and ubiquitination accessibility of SMARCA2-VHL conformations. | | | |

**Table S7:** Clustering statistics and docking energy scores of representative SMARCA2-VHL conformations.

| **Cluster ID** | **Size** | **Average Pairwise RMSD** | **Representative  SMARCA2VHL Conformation** | **Representative S Docking Score (Kcal/mol)** |
| --- | --- | --- | --- | --- |
| 1 | 41 | 4.40 | M1_P-P20 | -50.40 |
| 8 | 35 | 4.05 | M6_P-P392 | -34.61 |
| 6 | 27 | 5.04 | M4_P-P247 | -37.58 |
| 2 | 24 | 4.12 | M2_P-P41 | -47.26 |
| 5 | 11 | 3.74 | M5_P-P308 | -36.24 |
| 7 | 7 | 3.66 | M8_P-P609 | -30.83 |
| 4 | 6 | 3.53 | M3_P-P233 | -37.89 |
| 3 | 2 | 2.74 | M7_P-P421 | -34.17 |

| - **Cα RMSD of M4_P-P247 fitting on:** - **PDB Structure 7Z76=2.90 Å** | - **Cα RMSD of M5_P-P308 fitting on:** - **PDB Structure 7Z6L=4.90 Å** | - **Cα RMSD of M6_P-P392 fitting on:** - **PDB Structure 6HAX=3.70 Å** - **PDB Structure 6HAY=3.30 Å** - **PDB Structure 7S4E=3.50 Å** - **PDB Structure 7Z77=4.90 Å** |
| --- | --- | --- |

|  |  |  |
| --- | --- | --- |
|  |  |  |
|  |  |  |
|  |  |  |
|  |  |  |
|  |  |  |
|  |  |  |
|  |  |  |
| **Figure S18:** Cα RMSD, attachment atom distances and ubiquitination accessibility of SMARCA2-VHL conformations over 100 ns MD simulations. The red X indicates that the model was excluded from the attachment-atom distance monitoring due to its ubiquitination inaccessibility. | | |

- - 1. SMARCA4VHL

|  | |  | |
| --- | --- | --- | --- |
|  | |  | |
|  |  |  |  |
|  |  |  |  |
|  |  |  |  |
| **Figure S19:** Docking, filtering, clustering and ubiquitination accessibility of SMARCA4-VHL conformations. | | | |

**Table S8:** Clustering statistics and docking energy scores of representative SMARCA4-VHL conformations.

| **Cluster ID** | **Size** | **Average Pairwise RMSD** | **Representative  SMARCA4VHL Conformation** | **Representative S Docking Score (Kcal/mol)** |
| --- | --- | --- | --- | --- |
| 6 | 55 | 5.80 | M3_P-P77 | -43.61 |
| 9 | 48 | 5.01 | M5_P-P103 | -42.13 |
| 8 | 40 | 4.15 | M1_P-P6 | -56.68 |
| 3 | 23 | 4.66 | M4_P-P88 | -42.71 |
| 4 | 13 | 4.81 | M6_P-P132 | -41.09 |
| 2 | 9 | 4.55 | M8_P-P545 | -33.09 |
| 7 | 6 | 1.88 | M7_P-P133 | -41.09 |
| 1 | 5 | 4.92 | M2_P-P46 | -45.32 |
| 10 | 5 | 4.98 | M9_P-P560 | -32.84 |
| 5 | 3 | 0.82 | M10_P-P860 | -27.76 |

| - **Cα RMSD of M5_P-P103 fitting on:** - **PDB Structure 8G1Q = 4.60 Å** |
| --- |

|  |  |  |
| --- | --- | --- |
|  |  |  |
|  |  |  |
|  |  |  |
|  |  |  |
|  |  |  |
|  |  |  |
|  |  |  |
| **Figure S20:** Cα RMSD, attachment atom distances and ubiquitination accessibility of SMARCA4-VHL conformations over 100 ns MD simulations. The red X indicates that the model was excluded from the attachment-atom distance monitoring due to its ubiquitination inaccessibility. | | |

- - 1. BclxL-VHL

|  |  | |
| --- | --- | --- |
|  |  | |
|  |  |  |
|  |  |  |
| **Figure S21:** Docking, filtering, clustering and ubiquitination accessibility of BclxL-VHL conformations. | | |

**Table S9:** Clustering statistics and docking energy scores of representative BclxL-VHL conformations.

| **Cluster ID** | **Size** | **Average Pairwise RMSD** | **Representative  BclxL2VHL Conformation** | **Representative S Docking Score (Kcal/mol)** |
| --- | --- | --- | --- | --- |
| 3 | 60 | 5.70 | M1_P-P68 | -47.74 |
| 1 | 5 | 3.93 | M2_P-P462 | -38.02 |
| 2 | 2 | 0.01 | M3_P-P701 | -35.00 |
| 4 | 1 | 0.00 | M4_P-P1425 | -33.23 |

| - **Cα RMSD of M1_P-P2 fitting on:** - **PDB Structure 8FY0 = 4.90 Å** | - **Cα RMSD of M1_P-P2 fitting on:** - **PDB Structure 6ZHC = 3.90 Å** |
| --- | --- |

|  |  |  |
| --- | --- | --- |
|  |  |  |
|  |  |  |
|  |  |  |
| **Figure S22:** Cα RMSD, attachment atom distances and ubiquitination accessibility of BclxL-VHL conformations over 100 ns MD simulations. The red X indicates that the model was excluded from the attachment-atom distance monitoring due to its ubiquitination inaccessibility.  **Note:** In M4_P-P1425, distance was monitored for the attachment atoms of PROTAC6 (PDB ID: 6ZHC) after docking the corresponding warhead | | |

- - 1. BCL2-VHL

|  | |  | |
| --- | --- | --- | --- |
|  | |  | |
|  | |  |  |
|  |  |  |  |
| **Figure S23:** Docking, filtering, clustering and ubiquitination accessibility of BCL2-VHL conformations. | | | |

**Table S10:** Clustering statistics and docking energy scores of representative BCL2-VHL conformations

| **Cluster ID** | **Size** | **Average Pairwise RMSD** | **Representative  BCL2VHL Conformation** | **Representative S Docking Score (Kcal/mol)** |
| --- | --- | --- | --- | --- |
| 3 | 38 | 5.36 | M1_P-P78 | -46.51 |
| 2 | 9 | 4.11 | M5_P-P757 | -27.33 |
| 5 | 9 | 5.61 | M4_P-P419 | -34.74 |
| 1 | 3 | 3.49 | M2_P-P258 | -38.86 |
| 4 | 2 | 2.22 | M3_P-P347 | -36.42 |

| - **Cα RMSD of M4_P-P419 fitting on:** - **PDB Structure 8FY1 = 4.00 Å** | - **Cα RMSD of M5_P-P757 fitting on:** - **PDB Structure 8FY2 = 5.00 Å** |
| --- | --- |

|  |  |  |
| --- | --- | --- |
|  |  |  |
|  |  |  |
|  |  |  |
| **Figure S24:** Cα RMSD, attachment atom distances and ubiquitination accessibility of BCL2-VHL conformations over 100 ns MD simulations. The red X indicates that the model was excluded from the attachment-atom distance monitoring due to its ubiquitination inaccessibility. | | |

- - 1. WDR5-VHL

|  | |  | |
| --- | --- | --- | --- |
|  | |  | |
|  |  |  |  |
|  |  |  |  |
| \| **Figure S25:** Docking, filtering, clustering and ubiquitination accessibility of WDR5-VHL conformations. \| \| --- \| | | | |

**Table S11:** Clustering statistics and docking energy scores of representative WDR5-VHL conformations

| **Cluster ID** | **Size** | **Average Pairwise RMSD** | **Representative  WDR5VHL Conformation** | **Representative S Docking Score (Kcal/mol)** |
| --- | --- | --- | --- | --- |
| 4 | 64 | 5.50 | M4_P-P137 | -42.22 |
| 5 | 33 | 3.17 | M7_P-P364 | -36.00 |
| 3 | 24 | 3.20 | M2_P-P105 | -43.85 |
| 2 | 23 | 4.62 | M1_P-P2 | -55.15 |
| 1 | 13 | 2.68 | M3_P-P124 | -43.20 |
| 7 | 10 | 4.55 | M5_P-P234 | -39.44 |
| 6 | 6 | 3.81 | M6_P-P316 | -37.61 |

| - **Cα RMSD of M2_P-P105 fitting on:** - **PDB Structure 7JTP = 3.90 Å** | - **Cα RMSD of M4_P-P137 fitting on:** - **PDB Structure 7JTO = 2.30 Å** - **PDB Structure 8BB2 = 3.60 Å** - **PDB Structure 8BB3 = 2.50 Å** - **PDB Structure 8BB4 = 3.50 Å** - **PDB Structure 8BB5 = 3.50 Å** |
| --- | --- |

|  |  |  |
| --- | --- | --- |
|  |  |  |
|  |  |  |
|  |  |  |
|  |  |  |
|  |  |  |
| **Figure S26:** Cα RMSD, attachment atom distances and ubiquitination accessibility of WDR5-VHL conformations over 100 ns MD simulations. The red X indicates that the model was excluded from the attachment-atom distance monitoring due to its ubiquitination inaccessibility. | | |

- - 1. BRD4BD1-VHL

|  | |  | |
| --- | --- | --- | --- |
|  | |  | |
|  |  |  |  |
|  |  |  |  |
|  |  |  |  |
| **Figure S27:** Docking, filtering, clustering and ubiquitination accessibility of BRD4BD1-VHL conforamtions. | | | |

**Table S12:** Clustering statistics and docking energy scores of representative BRD4BD1-VHL conformations

| **Cluster ID** | **Size** | **Average Pairwise RMSD** | **Representative  BRD4BD1VHL Conformation** | **Representative S Docking Score (Kcal/mol)** |
| --- | --- | --- | --- | --- |
| 2 | 39 | 4.42 | M1_P-P4 | -61.58 |
| 4 | 15 | 5.09 | M6_P-P272 | -37.80 |
| 5 | 12 | 4.59 | M2_P-P27 | -49.08 |
| 1 | 11 | 3.05 | M3_P-P68 | -45.51 |
| 3 | 8 | 3.22 | M4_P-P86 | -44.50 |
| 8 | 4 | 3.52 | M8_P-P413 | -34.03 |
| 6 | 2 | 2.28 | M7_P-P404 | -34.25 |
| 7 | 2 | 3.05 | M5_P-P107 | -43.27 |
| 9 | 1 | 0.00 | M9_P-P611 | -27.61 |

| - **Cα RMSD of M4_P-P86 fitting on:** - **PDB Structure 7KHH = 4.60 Å** - **PDB Structure 8BDS = 4.70 Å** - **PDB Structure 8BEB = 4.80 Å** |
| --- |

|  |  |  |
| --- | --- | --- |
|  |  |  |
|  |  |  |
|  |  |  |
|  |  |  |
|  |  |  |
|  |  |  |
|  |  |  |
| **Figure S28:** Cα RMSD, attachment atom distances and ubiquitination accessibility of BRD4BD1-VHL conformations over 100 ns MD simulations. The red X indicates that the model was excluded from the attachment-atom distance monitoring due to its ubiquitination inaccessibility. | | |

- - 1. BRD4BD2-VHL

|  | |  | |
| --- | --- | --- | --- |
|  | |  | |
|  |  |  |  |
| **Figure S29:** Docking, filtering, clustering and ubiquitination accessibility of BRD4BD2-VHL conformations. | | | |

**Table S13:** Clustering statistics and docking energy scores of representative BRD4BD2-VHL conformations

| **Cluster ID** | **Size** | **Average Pairwise RMSD** | **Representative  BRD4BD2VHL Conformation** | **Representative S Docking Score (Kcal/mol)** |
| --- | --- | --- | --- | --- |
| 2 | 509 | 3.99 | M1_P-P55 | -45.32 |
| 3 | 5 | 5.11 | M3_P-P484 | -30.32 |
| 1 | 3 | 4.59 | M2_P-P340 | -34.68 |

| - **Cα RMSD of M2_P-P340 fitting on:** - **PDB Structure 5T35 = 1.90 Å** - **PDB Structure 7ZNT = 1.30 Å** - **PDB Structure 8BDT = 1.30 Å** - **PDB Structure 8BDX = 1.30 Å** |
| --- |

|  |  |  |
| --- | --- | --- |
|  |  |  |
|  |  |  |
| **Figure S30:** Cα RMSD, attachment atom distances and ubiquitination accessibility of BRD4BD2-VHL conformations over 100 ns MD simulations. The red X indicates that the model was excluded from the attachment-atom distance monitoring due to its ubiquitination inaccessibility. | | |

- - 1. KRas-VHL

|  |  | |
| --- | --- | --- |
|  |  | |
|  |  |  |
|  |  |  |
| **Figure S31:** Docking, filtering, clustering and ubiquitination accessibility of KRas-VHL conformations. | | |

**Table S14:** Clustering statistics and docking energy scores of representative KRas-VHL conformations

| **Cluster ID** | **Size** | **Average Pairwise RMSD** | **Representative  KRasVHL Conformation** | **Representative S Docking Score (Kcal/mol)** |
| --- | --- | --- | --- | --- |
| 1 | 3 | 4.50 | M3_P-P116 | -36.90 |
| 2 | 1 | 0.00 | M2_P-P21 | -45.55 |
| 3 | 1 | 0.00 | M1_P-P4 | -54.75 |
| 4 | 1 | 0.00 | M4_P-P368 | -24.55 |

| - **Cα RMSD of M4_P-P368 fitting on:** - **PDB Structure 8QW6 = 12.00 Å** - **PDB Structure 8QW7 = 11.70 Å** |
| --- |

|  |  |  |
| --- | --- | --- |
|  |  |  |
|  |  |  |
| **Figure S32:** Cα RMSD, attachment atom distances and ubiquitination accessibility of KRas-VHL conformations over 100 ns MD simulations. The red X indicates that the model was excluded from the attachment-atom distance monitoring due to its ubiquitination inaccessibility. | | |

- - 1. FAK-VHL

|  | |  | |
| --- | --- | --- | --- |
|  | |  | |
|  |  |  |  |
|  |  |  |  |
| **Figure S33:** Docking, filtering, clustering and ubiquitination accessibility of FAK-VHL conformations. | | | |

**Table S15:** Clustering statistics and docking energy scores of representative FAK-VHL conformations

| **Cluster ID** | **Size** | **Average Pairwise RMSD** | **Representative  FAKVHL Conformation** | **Representative S Docking Score (Kcal/mol)** |
| --- | --- | --- | --- | --- |
| 5 | 8 | 3.06 | M1_P-P2 | -69.16 |
| 1 | 7 | 2.27 | M2_P-P36 | -51.73 |
| 4 | 6 | 4.44 | M5_P-P189 | -43.34 |
| 3 | 5 | 4.01 | M4_P-P169 | -43.87 |
| 6 | 4 | 4.60 | M3_P-P43 | -51.05 |
| 2 | 3 | 3.67 | M6_P-P649 | -32.01 |

| - **Cα RMSD of M5_P-P189 fitting on:** - **PDB Structure 7PI4 = 3.10 Å** |
| --- |

|  |  |  |
| --- | --- | --- |
|  |  |  |
|  |  |  |
|  |  |  |
|  |  |  |
|  |  |  |
| **Figure S34:** Cα RMSD, attachment atom distances and ubiquitination accessibility of FAK-VHL conformations over 100 ns MD simulations. The red X indicates that the model was excluded from the attachment-atom distance monitoring due to its ubiquitination inaccessibility. | | |

- - 1. WEE1-VHL

|  |  | |
| --- | --- | --- |
|  |  | |
|  |  |  |
|  |  |  |
| **Figure S35:** Docking, filtering, clustering and ubiquitination accessibility of WEE1-VHL conformations. | | |

**Table S16:** Clustering statistics and docking energy scores of representative WEE1-VHL conformations.

| **Cluster ID** | **Size** | **Average Pairwise RMSD** | **Representative  WEE1VHL Conformation** | **Representative S Docking Score (Kcal/mol)** |
| --- | --- | --- | --- | --- |
| 2 | 42 | 4.77 | M1_P-P15 | -63.15 |
| 1 | 32 | 4.44 | M2_P-P95 | -52.08 |
| 3 | 9 | 3.42 | M4_P-P245 | -45.96 |
| 4 | 9 | 3.94 | M3_P-P96 | -52.05 |

| - **Cα RMSD of M2_P-P95 fitting on:** - **PDB Structure 8WDK = 3.50 Å** |
| --- |

|  |  |  |
| --- | --- | --- |
|  |  |  |
|  |  |  |
| **Figure S36:** Cα RMSD, attachment atom distances and ubiquitination accessibility of WEE1-VHL conformations over 100 ns MD simulations. The red X indicates that the model was excluded from the attachment-atom distance monitoring due to its ubiquitination inaccessibility. | | |

1. **POI-E3 Conformational Sampling Convergence Analysis**
   1. Cluster Saturation Analysis

|  |  | |  |
| --- | --- | --- | --- |
|  |  | |  |
|  |  | |  |
|  |  | |  |
|  | |  | |
| **Figure S37:** Cluster saturation analysis of all POI-E3 conformational ensembles. | | | |

- 1. Cluster Population Analysis

|  |  | |  |
| --- | --- | --- | --- |
|  |  | |  |
|  |  | |  |
|  |  | |  |
|  | |  | |
| **Figure S38:** Cluster population analysis of all POI-E3 conformational ensembles. | | | |

1. **Modeling of WEE1 and PKMYT1 CRBN-mediated PROTACs**
   1. Pharmacophore Features Guiding PROTAC Induced-fit docking

|  |
| --- |
|  |
| **Figure S39:** Pharmacophore features used to guide the placement of WEE1 and PKMYT1 PROTACs in the modelled WEE1-CRBN and PKMYT1-CRBN complexes. |

- 1. **PROTAC-Protein Interaction occupancy rates over 1 µs MD Simulations**
     1. ZNL-02-012, ZNL-02-040 and ZNL-02-047 in WEE1CRBN-M3

|  |
| --- |
|  |
|  |
| **Figure S40:** Schematic representation of detailed ZNL-02-012, ZNL02-040 and ZNL-02-047 interactions with WEE1-CRBN–M3 residues over 1 µs MD simulations. |

- - 1. ZNL-02-096 and TL12-186 in WEE1CRBN-M5

|  |
| --- |
|  |
| **Figure S41:** Schematic representation of detailed ZNL-02-096 and TL12-186 interactions with WEE1-CRBN–M5 residues over 1 µs MD simulations. |

- - 1. D16-M1P2 in PKMYT1CRBN-M4

|  |
| --- |
|  |
| **Figure S42**: Schematic representation of detailed D16-M1P2 interactions in the R and S configurations of the linker with PKMYT1-CRBN–M4 residues over 1 µs MD simulations. |

- 1. Ubiquitination Accessibility of Modelled ternary complexes over 1 µs MD Simulations
     1. MA071, ZNL-02-012, ZNL-02-040 and ZNL-02-047 in WEE1CRBN-M3

|  |
| --- |
|  |
|  |
|  |
| **Figure S43:** Ubiquitination accessibility heatmaps of MA071, ZNL-02-012, ZNL-02-040 and ZNL-02-047 in WEE1-CRBN–M3 over 1 µs MD simulations. |
|  |

- - 1. ZNL-02-096 and TL12-186 in WEE1CRBN-M5

|  |
| --- |
|  |
| **Figure S44:** Ubiquitination accessibility heatmaps of ZNL-02-096 and TL12-186 in WEE1-CRBN–M5 over 1 µs MD simulations. |

- - 1. D16-M1P2 in PKMYT1CRBN-M4

|  |
| --- |
|  |
| **Figure S45:** Ubiquitination accessibility heatmaps of D16-M1P2 in the R and S configurations of the linker in PKMYT1-CRBN–M4 over 1 µs MD simulations |

- 1. Attachment Atom Distances to Guide Linker Design

|  |  |  |
| --- | --- | --- |
|  | |  |
|  | |  |
| **Figure S46**: Attachment atom distances of WEE1-CRBN models monitored over 100ns MD simulations | | |

|  |  |  |
| --- | --- | --- |
|  | |  |
|  | |  |
|  | |  |
| **Figure S47**: Attachment atom distances of PKMYT1-CRBN models monitored between Compound 41 and Lenalidomide over 100ns MD simulations | | |

|  |  |  |
| --- | --- | --- |
|  | |  |
|  | |  |
| **Figure S48**: Attachment atom distances of PKMYT1-CRBN models monitored between Compound 4 and D6 over 100ns MD simulations | | |

1. **Synthesis of PROTACs HI100-103**

**Scheme 1**. Synthesis of intermediates **3**, **5**, **7** and **8**. Reagents and conditions: (a) LiHMDS, Dry THF, RT, 2h, Arg, 78%. (b) NaH, propanedinitrile, Pd(dppf)Cl_2_, Dry DME, 100 ᵒC, 3h, 81%. (c) NaOH, H_2_O_2_, 0 ᵒC, DMSO, 76%. (d) BBr_3_, Dry DCM, -70 ᵒC, 2h, 92 %. (e) Pd(dppf)Cl_2_.DCM (5 mol%), Pd (OAc)2 (10 mol %) Cs_2_CO_3_ (3 eq), dioxane/H_2_O (3:1), 80 ᵒC, 6 h, innert atom., 82%. (f) H_2_, MeOH/THF (1:1), 5h. (g) TFA, DCM, 0 ᵒC, 65%.

**Scheme 2**. Synthesis of the final PROTACs **HI100-103**. Reagents and conditions: (a) HATU, DIPEA, DMF, RT, 1h.

- 1. General Procedures for the Chemical Synthesis:

All materials and reagents were purchased from Sigma-Aldrich Co., Ltd. (Darmstadt, Germany) and abcr GmbH (Karlsruhe, Germany) and BLD Pharma. All solvents were analytically pure. Thin-layer chromatography was carried out on aluminium sheets coated with silica gel 60 F254 (Merck, Darmstadt, Germany). For medium-pressure liquid chromatography (MPLC)

Intermediates Biotage SNAP ultra-HP-sphere 25 μm columns containing silica gel were used. Dichloromethane (DCM): methanol (MeOH) and n-heptane: ethyl acetate mixtures were used as elution systems for MPLC. In the preparative high-pressure liquid chromatography used for purification of several PROTACs, LiChrosorb® RP-18 (7 μm) 250-25 Merck (Merck, Darmstadt, Germany) column was used. The applied mobile phase was a gradient with increasing polarity composed of acetonitrile/water/formic acid. HPLC purity was measured by UV absorbance at 254 nm using MeOH/H2O/0.05%TFA. The HPLC consisted of a LiChrosorb® RP-18 (5 μm) 100-4.6 Merck column (Merck, Darmstadt, Germany), two LC-10AD pumps, an SPD-M10A VP PDA detector, and a SIL-HT autosampler, all from the manufacturer Shimadzu (Kyoto, Japan). The absorption spectra were recorded with an SPD-M10A diode array detector Shimadzu spectrophotometer (Kyoto, Japan). Mass spectrometry was measured on an Advion expression CMS (Advion Interchim Scientific, Ithaca, NY, USA). ^1^H and ^13^C NMR spectra were taken on a Varian Inova 400 using deuterated DMSO as solvent. Chemical shifts were referenced to the residual solvent signals. The following abbreviations and formulas for solvents and reagents were used: ethyl acetate (EtOAc), *N*,*N*-dimethylformamide (DMF), dimethyl sulfoxide (DMSO), methanol (MeOH), tetrahydrofuran (THF), water (H_2_O), dichloromethane (DCM), *N*,*N* diisopropylethylamine (DIPEA), O-(7-azabenzotriazol-1-yl)-*N*, *N*, *N*′, *N*′-tetramethyluroniumhexafluorphosphate (HATU) and hydrochloric acid (HCl), trifluoroacetic acid (TFA), Dimethoxy ethane (DME). Intermediates **9-12** were prepared and characterized as reported [1-3].

- 1. Synthesis of 3,5-dibromo-*N*-(3-methoxy-2,6-dimethylphenyl)pyridin-2-amine (**3**).

Li bis(trimethylsilyl)amide (LiHMDS) (1.5 M in THF, 6.63 ml, 11.77 mmol) was added dropwise under Arg. and 0 ᵒC to a solution of 3,5-dibromo-2-fluoropyridine(**1**) (1.50g, 5.89 mmol) and 3-methoxy-2,6-dimethylaniline (**2**) (0.75g, 5.89 mmol) and the mixture was stirred at room temprature for 2 h. The completion oft he reaction was checked by TLC (EtAc/Hept 3:1). The reaction was quenched with water then extracted with EtAc. The crude material after evaporation was submitted to purification using MPLC using gradient elution with (EtOAc/Hept) from 1 to 4% to obtain 3,5-dibromo-*N*-(3-methoxy-2,6-dimethylphenyl)pyridin-2-amine (**3**) (1.97g, 87%). ^1^H NMR (402 MHz, DMSO-*d_6_*) *δ* 8.05 (d, *J* = 2.1 Hz, 1H), 7.93 (d, *J* = 1.9 Hz, 2H), 7.01 (d, *J* = 8.4 Hz, 1H), 6.78 (d, *J* = 8.4 Hz, 1H), 3.74 (s, 3H), 1.98 (s, 3H), 1.89 (s, 3H). MS m/z: 387.1 [M + H]^+^ .

- 1. Synthesis of 2-amino-5-bromo-1-(3-methoxy-2,6-dimethylphenyl)-1*H*-pyrrolo[2,3-*b*]pyridine-3-carbonitrile (**4**).

NaH (60% dispersion in mineral oil, 0.60 g, 16.78 mmol) suspension in DME (20 mL) was added to a solution of malononitrile (0.91 g, 15.39 mmol) in DME (10 mL) at 0 ᵒC then the mixture was stirred at RT for 1 h. The solution was degassed and purged 3 times with Argon then compound **3** (2.70 g, 6.99 mmol) followed by Pd(dppf)Cl_2_ (0.46 g. 10 mol%) was added and the temperature was elevated at 100 ᵒC for 3 h. After disappearance of the starting materials in TLC (EtAC: Hept. 1:1). The reaction mixture was quenched by water and the extracted with EtOAc then dried and purified using MPLC (EtAc: Hept, 28%) to obtain the target compound (2.1g, 81%). Analytical data coincide with reported data [4].

- 1. Synthesis of 2-amino-5-bromo-1-(3-hydroxy-2,6-dimethylphenyl)-1*H*-pyrrolo[2,3-*b*]pyridine-3-carboxamide (**5)**.

Compound **4** (0.67 g, 1.8 mmol) was dissolved in DMSO and then 2 mL 2M NaOH (4 mmol) was added at 0 ᵒC followed by H_2_O_2_ (5 ml, 30%, 40 mmol) and stirring for 30 min. Water was added to quench the reaction then extract with EtOAc, dried and purified using MPLC (EtOAc: Hept, 45%). The obtained product (0.53 mg, 1.36 mmol) was dissolved in dry DCM then purged with Argon three times followed by addition of 1M BBr_3_ (1.71 g, 6.81 mmol) 1under -70 ᵒC. Stirring of the mixture was continued after RT for 2 h. The reaction was quenched by addition of 20 mL MeOH slowly under 0 ᵒC. Water was added to the mixture and the organic layer was separated. The aqueous layer was washed with EtOAc and the combined organic layer was dried, evaporated and obtained pure enough for the next step. Analytical data of compound **5** were found as previously reported [4].

- 1. Synthesis of *tert*‐butyl 4‐[2‐amino‐3‐carbamoyl‐1‐(3‐hydroxy‐2,6‐dimethylphenyl) ‐1*H*‐pyrrolo[2,3‐b]pyridin‐5‐yl]‐1,2,3,6‐tetrahydropyridine‐1‐carboxylate (**7)**.

Solution of 2-amino-5-bromo-1-(3-hydroxy-2,6-dimethylphenyl)-1*H*-pyrrolo[2,3-b]pyridine-3-carboxamide (**5**) (0.37 g, 0.99 mmol), *tert*-butyl 4-(4,4,5,5-tetramethyl-1,3,2-dioxaborolan-2-yl)-3,6-dihydropyridine-1(2*H*)-carboxylate (**6**) (0.34 g, 1.08 mmol), Pd(OAc)_2_ (11.07 mg, 0.05 mmol), Pd(dppf)Cl_2_ (72.15 mg, 0.09 mmol), and Cs_2_CO_3_ (0.96 g, 2.96 mmol) in 1,4-dioxane:water (10 mL, 3:1 mixture) was heated to 80 oC for 6 h under Argon atmosphere. The reaction was cooled to room temperature, diluted with EtOAc (30 mL) filtered through celite, and concentrated under vacuum. The residue was purified by MPLC (DCM: MeOH). The gradient system starts from 1% till 5% and the obtained yield was (0.25g, 53%). ^1^H NMR (402 MHz, DMSO-*d_6_*) *δ* 9.45 (s, 1H), 7.98 (d, J = 2.0 Hz, 1H), 7.80 (d, J = 1.9 Hz, 1H), 7.03 (d, J = 8.3 Hz, 1H), 6.94 (s, 2H), 6.88 (d, J = 8.3 Hz, 1H), 6.78 (s, 2H), 6.13 (s, 1H), 3.98 (d, J = 3.8 Hz, 2H), 3.53 (t, J = 5.7 Hz, 2H), 2.53 (q, J = 5.4 Hz, 2H), 1.72 (s, 3H), 1.64 (s, 3H), 1.41 (s, 9H). MS m/z: 478.1 [M + H]^+^ .

- 1. Synthesis of 4-[2-amino-3-carbamoyl-1-(3-hydroxy-2,6-dimethylphenyl)-1*H*-pyrrolo[2,3-b]pyridin-5-yl]piperidin-1-ium trifluoroacetate **8**.

Compound (**7**) (0.25 g, 0.48 mmol) was dissolved in a mixture of MeOH/THF (1:1) followed by addition of Pd/C 10% (26.0 mg, 0.03 mmol). The mixture was purged with Argon then H_2_ with gentle stirring for 6h. The reaction mixture was filtered through celite, and concentrated under vacuum. The obtained product (0.18g, 97%) was proceeded to the next reaction without purification. The residue was dissolved in DCM and 5 mL TFA was added at 0 ᵒC. The mixture was stirred for 1 h at RT then the solvent was evaporated and the residue purified by MPLC (DCM: MeOH) and the obtained yield for compound (**8**) was (0.13g, 93%). ^1^H NMR (402 MHz, DMSO-*d_6_*) *δ* 9.45 (s, 1H), 7.89 (d, J = 1.9 Hz, 1H), 7.62 (d, J = 1.8 Hz, 1H), 7.02 (d, J = 8.3 Hz, 1H), 6.88 (d, J = 8.0 Hz, 3H), 6.74 (s, 2H), 4.12 – 4.04 (m, 2H), 2.77 (s, 2H), 2.67 – 2.63 (m, 2H), 1.71 (m, 6H), 1.63 (s, 3H). MS m/z: 380.3 [M + H]^+^ .

- 1. General procedure for synthesis of the final PROTACs **HI100-103**.

A solution of the appropriate CRBN ligands **9**-**12** (0.14 mmol), HATU (61.9 mg 0.16 mmol), and DIPEA (52.6 mg 0.41 mmol) in DMF (5 mL) was stirred at RT for 10 min., then compound (**8**) (80 mg, 0.16 mmol) was added. The reaction mixture was stirred at RT for 1 h. After completion of the reaction as indicated by TLC, Brine was added, and the mixture was extracted using EtOAc. The combined organic layer was washed with an aqueous 1 M ammonium chloride solution, followed by an aqueous 1 M sodium bicarbonate solution and brine. The combined organic extract was dried over anhydrous sodium sulfate, the organic layer was filtered, then evaporated under reduced pressure to yield the crude amide, which was purified using MPLC using DCM : MeOH (3-10% MeOH).

- - 1. 2‐Amino‐5‐(1‐{1‐[2‐(2,6‐dioxopiperidin‐3‐yl)‐1,3‐dioxo‐2,3‐dihydro‐1*H*‐isoindol‐5‐yl] piperidine‐4‐carbonyl}piperidin‐4‐yl)‐1‐(3‐hydroxy‐2,6‐dimethylphenyl)‐1*H*‐pyrrolo[2,3‐b]pyridine‐3‐carboxamide (**HI100**).

^1^H NMR (600 MHz, DMSO-*d_6_*) *δ* 11.04 (s, 1H), 9.44 (s, 1H), 7.89 (d, *J* = 1.9 Hz, 1H), 7.67 – 7.61 (m, 2H), 7.31 (d, *J* = 2.2 Hz, 1H), 7.23 (dd, *J* = 8.7, 2.3 Hz, 1H), 7.02 (d, *J* = 8.3 Hz, 1H), 6.88 (d, *J* = 8.4 Hz, 3H), 6.74 (s, 2H), 5.04 (dd, *J* = 12.9, 5.4 Hz, 1H), 4.57 (d, *J* = 12.8 Hz, 1H), 4.16 (d, *J* = 13.1 Hz, 1H), 4.05 (d, *J* = 12.8 Hz, 2H), 3.13 – 2.97 (m, 4H), 2.93 – 2.73 (m, 2H), 2.60 – 2.49 (m, 2H), 2.07 – 1.93 (m, 1H), 1.85 (d, *J* = 12.9 Hz, 1H), 1.67 (d, *J* = 33.1 Hz, 14H). ^13^C NMR (151 MHz, DMSO-*d_6_*) *δ* 173.23, 172.37, 170.52, 168.78, 168.06, 167.41, 155.31, 154.64, 153.25, 144.90, 137.64, 134.76, 134.52, 132.78, 128.22, 127.39, 125.47, 124.37, 122.02, 118.49, 118.08, 118.04, 115.99, 108.24, 83.73, 55.34, 49.21, 47.21, 45.97, 42.34, 37.41, 34.57, 33.36, 31.43, 28.07, 27.94, 22.65, 17.33, 11.32. (ESI-MS) [M+Na]^+^ : m/z = 769,1 [M+H]^+^ : m/z = 747.1, HPLC: rt 11.63 min (purity 99.04%)

- - 1. 2-Amino-5-(1-{6-[2-(2,6-dioxopiperidin-3-yl)-1-oxoisoindolin-5-yl]hex-5-ynoyl}piperidin-4-yl)-1-(3-hydroxy-2,6-dimethylphenyl)-1*H*-pyrrolo[2,3-b]pyridine-3-carboxamide (**HI101**).

^1^H NMR (600 MHz, DMSO-*d_6_*) *δ* 10.95 (s, 1H), 9.45 (s, 1H), 7.89 (d, *J* = 1.9 Hz, 1H), 7.69 – 7.60 (m, 3H), 7.50 (dd, *J* = 7.9, 1.4 Hz, 1H), 7.02 (d, *J* = 8.3 Hz, 1H), 6.88 (d, *J* = 8.2 Hz, 3H), 6.74 (s, 2H), 5.07 (dd, *J* = 13.3, 5.1 Hz, 1H), 4.58 (d, *J* = 12.8 Hz, 1H), 4.41 (dd, *J* = 17.6, 3.2 Hz, 1H), 4.28 (dd, *J* = 17.6, 3.6 Hz, 1H), 4.01 (d, *J* = 13.3 Hz, 1H), 3.09 (t, *J* = 12.7 Hz, 1H), 2.95 – 2.68 (m, 2H), 2.64 – 2.51 (m, 3H), 2.37 (td, *J* = 13.2, 4.5 Hz, 1H), 2.00 – 1.93 (m, 1H), 1.81 (p, *J* = 7.1 Hz, 3H), 1.67 (d, *J* = 32.6 Hz, 13H). ^13^C NMR (151 MHz, DMSO-*d_6_*) *δ* 173.26, 171.35, 170.11, 168.77, 167.88, 154.64, 153.25, 144.89, 142.82, 137.63, 134.80, 132.77, 131.59, 131.38, 128.21, 127.39, 126.90, 126.82, 124.37, 123.59, 122.03, 118.48, 115.99, 93.27, 83.72, 81.06, 52.14, 47.50, 46.10, 42.22, 34.24, 33.30, 31.76, 31.65, 24.60, 22.87, 18.90, 17.32, 11.31. (ESI-MS) [M+Na]^+^ : m/z = 739,1, [M+H]^+^ : m/z = 717.1, HPLC: rt 11.69 min (purity 96.98%).

- - 1. 2-Amino-5-[1-(2-{4-[2-(2,6-dioxopiperidin-3-yl)-1,3-dioxoisoindolin-5-yl]piperazin-1-yl}acetyl)piperidin-4-yl]-1-(3-hydroxy-2,6-dimethylphenyl)-1*H*-pyrrolo[2,3-b]pyridine-3-carboxamide (**HI102**).

^1^H NMR (600 MHz, DMSO-*d_6_*) *δ* 11.04 (s, 1H), 9.44 (s, 1H), 7.95 – 7.84 (m, 1H), 7.71 – 7.60 (m, 2H), 7.36 (s, 1H), 7.29 – 7.22 (m, 1H), 7.02 (d, *J* = 8.3 Hz, 1H), 6.91 – 6.84 (m, 3H), 6.73 (s, 2H), 5.05 (dd, *J* = 12.8, 5.4 Hz, 1H), 4.54 (d, *J* = 12.6 Hz, 1H), 4.08 (s, 1H), 3.62 – 3.45 (m, 7H), 3.10 (t, *J* = 12.6 Hz, 1H), 2.87 (s, 1H), 2.86 – 2.77 (m, 1H), 2.71 (s, 1H), 2.60 – 2.50 (m, 3H), 2.06 (s, 3H), 2.04 – 1.96 (m, 1H), 1.86 – 1.79 (m, 2H), 1.67 (d, *J* = 49.4 Hz, 7H).^13^C NMR (151 MHz, DMSO-*d_6_*) *δ* 173.22, 170.48, 168.82, 167.96, 167.40, 162.77, 154.63, 153.20, 144.89, 137.44, 134.29, 132.76, 128.22, 127.38, 125.37, 124.36, 122.21, 118.51, 115.99, 83.75, 52.40, 49.26, 40.40, 40.26, 40.13, 39.99, 39.85, 39.71, 39.57, 36.23, 31.42, 31.23, 31.12, 22.62, 17.32, 11.31. (ESI-MS) [M+]+ : m/z = 746.8, HPLC: rt 9.65 min (purity 95.02%)

- - 1. 2-Amino-5-(1-{7-[2-(2,6-dioxopiperidin-3-yl)-1-oxoisoindolin-5-yl]hept-6-ynoyl}piperidin-4-yl)-1-(3-hydroxy-2,6-dimethylphenyl)-1*H*-pyrrolo[2,3-b]pyridine-3-carboxamide (**HI103**).

^1^H NMR (600 MHz, DMSO-*d_6_*) *δ* 10.96 (s, 1H), 9.44 (s, 1H), 7.89 (d, *J* = 1.9 Hz, 1H), 7.68 – 7.58 (m, 3H), 7.48 (dd, *J* = 7.9, 1.3 Hz, 1H), 7.02 (d, *J* = 8.3 Hz, 1H), 6.88 (d, *J* = 8.5 Hz, 3H), 6.74 (s, 2H), 5.08 (dd, *J* = 13.3, 5.1 Hz, 1H), 4.58 (d, *J* = 12.7 Hz, 1H), 4.41 (d, *J* = 17.4 Hz, 1H), 4.29 (d, *J* = 17.4 Hz, 1H), 4.04 – 3.98 (m, 1H), 3.08 (t, *J* = 12.8 Hz, 1H), 2.93 – 2.84 (m, 1H), 2.76 (t, *J* = 12.2 Hz, 1H), 2.62 – 2.53 (m, 2H), 2.52 – 2.49 (m, 2H), 2.45 – 2.31 (m, 3H), 2.02 – 1.95 (m, 1H), 1.78 – 1.56 (m, 14H).^13^C NMR (151 MHz, DMSO-*d_6_*) *δ* 173.26, 171.36, 170.53, 168.79, 167.88, 154.64, 153.24, 144.88, 142.83, 142.62, 137.61, 134.82, 132.77, 131.55, 131.33, 128.21, 127.40, 126.98, 126.90, 126.74, 124.38, 123.59, 122.05, 118.48, 116.00, 93.57, 83.73, 80.86, 52.15, 47.50, 46.14, 34.26, 33.35, 32.41, 31.65, 28.24, 24.67, 22.88, 19.02, 17.32, 11.31. (ESI-MS) [M+Na]^+^ : m/z = 751,9 [M+H]^+^ : m/z = 729,9 , HPLC: rt 12.25 min (purity 94.28%)

1. **Analytical charts of PROTACs HI100-103**
   1. Analytical charts of HI100

**Figure S49:** ^13^CNMR chart for compound HI100.

**Figure S50:** ^1^HNMR chart for compound HI100.

**Figure S51:** HPLC purity analysis for compound HI100.

- 1. Analytical charts of H101

**Figure S52:** ^13^CNMR chart for compound HI101.

**Figure S53**: ^1^HNMR chart for compound HI101.

**Figure S54:** HPLC purity analysis for compound HI101.

- 1. Analytical charts of H102

**Figure S55:** ^13^CNMR chart for compound HI102.

**Figure S56:** ^1^HNMR chart for compound HI102.

**Figure S57:** HPLC purity analysis for compound HI102.

- 1. Analytical charts of H103

**Figure S58:** ^13^CNMR chart for compound HI103.

**Figure S59:** ^1^HNMR chart for compound HI103.

**Figure S60:** HPLC purity analysis for compound HI103.

**References**

1. Ibrahim HS, Guo M, Hilscher S, Erdmann F, Schmidt M, Schutkowski M, Sheng C, Sippl W (2024) Probing class I histone deacetylases (HDAC) with proteolysis targeting chimera (PROTAC) for the development of highly potent and selective degraders. Bioorganic Chemistry 107887.‏ <https://doi.org/10.1016/j.bioorg.2024.107887>
2. Hu H, Wang Y, Wang M, Zhang Z, Gu X, Sun R, Liu X, Li N, Ding N, Li W, Zhao X, Li C, Huang Z, Wang X, Li X, Liu S, Yang S, Yang G (2025) Translational Research on the Oral Delivery of the Cytotoxic PROTAC Molecule via Tumor-Targeting Prodrug Strategy for Triple-Negative Breast Cancer Treatment. Journal of Medicinal Chemistry 20464-20486.‏ <https://doi.org/10.1021/acs.jmedchem.5c01640>
3. He P, Wen C, Zhang X, Yin H (2025) Discovery of a novel CRBN-recruiting cGAS PROTAC degrader for the treatment of ulcerative colitis. Journal of Medicinal Chemistry 5551-5572.‏ <https://doi.org/10.1021/acs.jmedchem.4c02774>
4. Szychowski J, Papp R, Dietrich E, Liu B, Vallée F, Leclaire ME, Fourtounis J, Martino G, Perryman AL, Pau V, Yin SY, Mader P, Roulston A, Truchon JF, Marshall CG, Diallo M, Duffy NM, Stocco R, Godbout C, Bonneau-Fortin A, Kryczka R, Bhaskaran V, Mao D, Orlicky S, Beaulieu P, Turcotte P, Kurinov I, Sicheri F, Mamane Y, Gallant M, Black WC (2022) Discovery of an orally bioavailable and selective PKMYT1 inhibitor, RP-6306. Journal of medicinal chemistry 10251-10284.‏ <https://doi.org/10.1021/acs.jmedchem.2c00552>
